# Programming the lifestyles of engineered bacteria for cancer therapy

**DOI:** 10.1093/nsr/nwad031

**Published:** 2023-02-14

**Authors:** Shengwei Fu, Rongrong Zhang, Yanmei Gao, Jiarui Xiong, Ye Li, Lu Pu, Aiguo Xia, Fan Jin

**Affiliations:** Hefei National Research Center for Physical Sciences at the Microscale; Department of Polymer Science and Engineering, University of Science and Technology of China, Hefei 230026, China; CAS Key Laboratory of Quantitative Engineering Biology, Shenzhen Institute of Synthetic Biology, Shenzhen Institutes of Advanced Technology, Chinese Academy of Sciences, Shenzhen 518055, China; Hefei National Research Center for Physical Sciences at the Microscale; Department of Polymer Science and Engineering, University of Science and Technology of China, Hefei 230026, China; Hefei National Research Center for Physical Sciences at the Microscale; Department of Polymer Science and Engineering, University of Science and Technology of China, Hefei 230026, China; CAS Key Laboratory of Quantitative Engineering Biology, Shenzhen Institute of Synthetic Biology, Shenzhen Institutes of Advanced Technology, Chinese Academy of Sciences, Shenzhen 518055, China; West China School of Medicine; West China Hospital of Sichuan University, Chengdu 610065, China; CAS Key Laboratory of Quantitative Engineering Biology, Shenzhen Institute of Synthetic Biology, Shenzhen Institutes of Advanced Technology, Chinese Academy of Sciences, Shenzhen 518055, China; Hefei National Research Center for Physical Sciences at the Microscale; Department of Polymer Science and Engineering, University of Science and Technology of China, Hefei 230026, China; CAS Key Laboratory of Quantitative Engineering Biology, Shenzhen Institute of Synthetic Biology, Shenzhen Institutes of Advanced Technology, Chinese Academy of Sciences, Shenzhen 518055, China

**Keywords:** genetic engineering, drug delivery, cancer therapy, optogenetics, synthetic biology

## Abstract

Bacteria can be genetically engineered to act as therapeutic delivery vehicles in the treatment of tumors, killing cancer cells or activating the immune system. This is known as bacteria-mediated cancer therapy (BMCT). Tumor invasion, colonization and tumor regression are major biological events, which are directly associated with antitumor effects and are uncontrollable due to the influence of tumor microenvironments during the BMCT process. Here, we developed a genetic circuit for dynamically programming bacterial lifestyles (planktonic, biofilm or lysis), to precisely manipulate the process of bacterial adhesion, colonization and drug release in the BMCT process, via hierarchical modulation of the lighting power density of near-infrared (NIR) light. The deep tissue penetration of NIR offers us a modality for spatio-temporal and non-invasive control of bacterial genetic circuits *in vivo*. By combining computational modeling with a high-throughput characterization device, we optimized the genetic circuits in engineered bacteria to program the process of bacterial lifestyle transitions by altering the illumination scheme of NIR. Our results showed that programming intratumoral bacterial lifestyle transitions allows precise control of multiple key steps throughout the BMCT process and therapeutic efficacy can be greatly improved by controlling the localization and dosage of therapeutic agents via optimizing the illumination scheme.

## Teaser

Programming intratumoral bacterial life style transitions allow precise control of multiple key steps throughout the bacteria-mediated cancer therapy process and therapeutic efficacy can be greatly improved by controlling the localization and dosage of therapeutic agents via optimizing the illumination scheme.

## INTRODUCTION

The hypoxia and immune-privileged tumor microenvironment is one of the biggest obstacles in the way of many cancer therapeutics [1–3], such as chemotherapy [4], radiotherapy [5] and immunotherapy [6,7]. Nevertheless, this unique microenvironment of solid tumors is ideal for the colonization and proliferation of a variety of obligate anaerobes and facultative anaerobes [8–11], including *Listeria, Clostridium* and *Salmonella*, which have been well studied and extensively used in the treatment of cancers. In addition to their inherent antitumor effects by stimulation of the innate immune system or secretion of natural antitumor products [12–15], bacteria were also engineered to act as therapeutic vehicles to deliver different payloads for improved *in situ* cancer therapy [16–22]. The engineered living therapeutics endowed with synthetic genetic circuits have shown advantages over conventional cancer therapies in terms of flexibility, specificity and predictability [23,24]. Bacteria-mediated cancer therapy (BMCT) has therefore emerged as a promising strategy for the treatment of cancers.

Tumor invasion, colonization and tumor regression are key biological events associated with antitumor effects in the process of BMCT [25]. Different approaches have been adopted to improve the therapeutic efficacy via manipulating these biological events, such as virulence attenuation [26], bacterial colonization enhancement [27,28] and drug release strategies [29–31]. For example, ${\rm{\Delta ppGpp}}$*S. typhimurium* strain, expressing tumor-specific ligands such as? arginine-glycine-aspartate (RGD) peptide on the cell surface, improves bacterial tumor targeting and therapeutic efficacy [28]. Although these strategies resulted in improved therapeutic efficacy in mice, the situation is different when it comes to clinical study, which shows lower colonization densities and greater heterogeneity [32]. For most therapeutic agents like chemical drugs and cytotoxic proteins used in BMCT, therapeutic efficacy is dose-dependent, requiring higher colonization for better therapeutic efficacy. These clinical trials, on the other hand, suggest that intratumoral colonization stems from the bidirectional biological interactions between bacteria and the host tumor microenvironment (TME), which is dynamic and uncontrollable to some extent [33].

As tumor treatment is a long-term process that requires controlled and sustained release of therapeutic agents into the TME [29,34], tumor regression is difficult to achieve by simply manipulating a certain biological event in BMCT, such as bacterial colonization and drug release. While the development of nanotechnology and the increased availability of versatile materials, including polymeric hydrogels and lipids, have opened up new possibilities for achieving sustained drug release [35], this remains a challenge in BMCT. Programmable lysis is a well-studied field and has demonstrated its high efficiency in protein release and medical applications [18,29,36,37]. Din and co-workers developed a synchronized lysis system that enables periodic drug production with the fluctuation in bacterial populations [29]. In this system, tumor growth is inhibited by the therapeutic agents released via bacterial lysis, while the process of bacterial colonization is uncontrollable and the density of bacterial colonization cannot exceed a threshold due to intrinsic lysis mechanism. Therefore, multiple injections are necessary to maintain the treatment for better therapeutic efficacy, but at the same time bring about pharmacological adverse effects.

Optogenetics allows the control of cellular signaling in real time and is recently being employed for therapeutic applications [38]. Using optogenetics, we developed a genetic circuit in engineered bacteria that allows dynamic manipulation of bacterial lifestyles (planktonic, biofilm and lysis lifestyle) to precisely control the process of bacterial adhesion, colonization and drug release, with near-infrared (NIR) light in the BMCT process. The deep tissue penetration of NIR enables spatio-temporal and non-invasive control of the genetic circuit [39,40] and is widely used to trigger certain behaviors of bacteria *in vivo* [41,42]. In addition, the lighting power density (LPD) of NIR used to program the lifestyles of engineered bacteria H017 shows a reduction of 3 orders of magnitude compared with that of bacteria-based photothermal therapy (PTT) [43,44], which enables widespread clinical use outside of dermatological indications. We also explored the potential of H017 for cancer therapy. Much less frequent injections of H017 can accomplish controllable drug release and tumor repression by dynamically programming the intratumoral bacterial lifestyle transitions via hierarchical modulation of the LPD of NIR. In all, the programmable lifestyle system enables multiple critical steps of the entire BMCT process to be precisely controlled and expands the strategy for enhanced bacterial colonization and sustained drug release in solid tumors.

## RESULTS

### Attenuation of *P. aeruginosa* for cancer therapy

*Salmonella typhimurium* and some other pathogens [45], which have been extensively studied for the treatment of tumors, have rarely been reported to colonize the lung. *Pseudomonas aeruginosa* is facultative anaerobe [46,47] that can not only naturally colonize the lung in mouse models when inoculated intranasally (Supplementary Table S1), but also preferentially accumulate in solid tumors [48,49] just like *Salmonella* and *E. coli* (Supplementary Table S2) when administrated systemically. In addition, *E. coli* and *S. typhimurium* are tended to colonize the necrotic part of the tumor but *P. aeruginosa* was even found in viable parts of the tumor [48]. This unique colonization preference may improve antitumor efficiency in solid tumors without a necrotic area. Besides, bacteriocins (exotoxin A, PE) produced by *P. aeruginosa* shows antitumor activities [50] and *P. aeruginosa* preparation (PAP) has already been used to inhibit the metastasis of lung cancer in clinical trials [51]. Taken together, we reasoned that *P. aeruginosa* has an advantage over other model bacteria in bacteria-mediated lung cancer therapy considering its intrinsic lung colonization capacity and it is noteworthy to further exploit it as novel chassis bacteria in BMCT.

Attenuation of human pathogens is critical in BMCT for safety concerns. For *P. aeruginosa*, Vfr (virulence factor regulator) is a cAMP-binding transcriptional regulator that controls the production of multiple virulence factors on a global level [52]. *Vfr* mutant showed notably weakened cytotoxicity to host cells [53]. In addition, Type III secretion system (T3SS) exoenzyme S (ExoS) and exoenzyme T (ExoT) are involved in *P. aeruginosa* pathogenesis. *Pseudomonas aeruginosa* mutant lacking both *exoS* and *exoT* remarkably decreases the ability to survive or spread systemically [54]. Based on previous studies, by triple deletion of *exoS, exoT* and *vfr* successively, we constructed a *P. aeruginosa* strain ExoST. ExoST showed significantly decreased cytotoxicity when co-cultured with A549 cells *in vitro* compared with the wild-type strain (Supplementary Fig. S1a–c). In addition, the survival ratio of mice challenged with subcutaneous (s.c.) injection of ExoST or wild-type strain was 100% or 12.5%, respectively (Supplementary Fig. S1d), indicating that ExoST is a highly attenuated *P. aeruginosa* strain *in vivo*. To further evaluate the attenuation efficacy, we conducted inflammatory factors tests and pathological observation of main organs and no significant adverse effects were observed (Supplementary Fig. S1f and g). Collectively, the attenuated *P. aeruginosa* strain ExoST could be adopted in the preliminary proof-of-concept in mouse models.

### Genetic circuit design for programming bacterial lifestyles

We designed three distinct lifestyles to implement different functions in the BMCT process. The adhesion of bacteria to tissue surfaces is an essential step in bacterial invasions in BMCT. Thus, we speculated that setting the initial bacterial lifestyle to non-colonization (planktonic lifestyle) prior to invading tumor tissues can greatly reduce its toxicity to normal tissues. It is well known that the formation of biofilm prevents bacteria from neutrophil-mediated killing [55] and facilitates their adhesion to the surface of various tissues [56]. We hypothesized that intratumoral biofilm formation of *P. aeruginosa* may contribute to immune privilege, enhanced tumor colonization and improved therapeutic efficacy in BMCT. So, the biofilm lifestyle was also designed. Extracellular polymeric substances (EPS) play a vital role in bacterial initial adhesion, surface colonization, biofilm formation and dispersal, and EPS production can be regulated by the bacterial secondary messenger c-di-GMP [57]. The intracellular c-di-GMP can be degraded by phosphodiesterase PA2133 and synthesized by BphS in a NIR-dependent manner [58]. Moreover, the LPD and illumination duration of NIR can be precisely manipulated *in vitro*. Therefore, with the genetic circuit consisting of a NIR sensor module and a c-di-GMP hydrolysis module, the bacterial intratumoral lifestyle can be tuned from planktonic (non-colonization) to biofilm (colonization) when irradiated with appropriate LPDs of NIR.

To facilitate drug release after bacterial colonization of tumors, the lysis lifestyle was designed as well. We first screened the lysis genes from different microorganisms (Supplementary Fig. S2 and Supplementary Table S3) to obtain a lysis cassette *LKD* that can cause lysis of wild-type *P. aeruginosa* PAO1 at a relatively low expression level, given that a low expression level of lysis gene can greatly reduce the burden of therapeutic bacteria and thus improve the stability of the inducible lysis system [59]. To avoid the leaky expression of the genes to induce the death of a small fraction of cells, the *LKD* was placed behind the terminator tR' (Supplementary Fig. S3), so that the *LKD* is activated by the production of antiterminator protein Q, which is driven by the c-di-GMP responsive promoter P*cdrA*. As the LPD increases, the production of Q protein reaches a threshold, leading to the lysis of bacteria and the release of antitumor drugs into the TME. Besides, the expression noise of *Q* contributes to phenotypic variability and results in asynchronous lysis behavior. When the LPD of NIR is tuned to a lower level, the surviving bacteria continue to grow as seeds, allowing the subsequent regulation of bacterial lifestyle transitions for sustainable drug synthesis and release.

**Figure 1.**
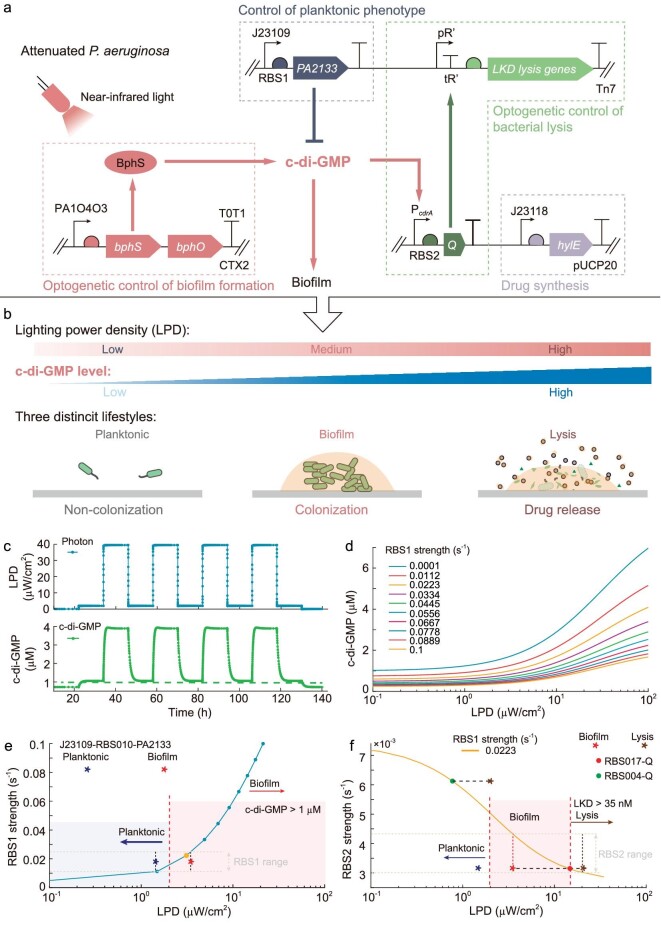
Genetic circuit design for programming bacterial lifestyles. (a) The programmable bacterial lifestyle system consists of four modules: the core NIR light-responsive module producing c-di-GMP and promoting biofilm formation (red); a lysis circuit module driven by a c-di-GMP-responsive promoter facilitating drug release (green); c-di-GMP hydrolysis module inhibiting bacteria from adhering to the surface (dark blue); and drug synthesis module to produce anticancer proteins (purple). (b) Schematic diagram of programmable manipulation of bacterial lifestyles using optogenetics. By adjusting the lighting power density (LPD) of near-infrared light (NIR), bacteria exhibit three different lifestyles: planktonic lifestyle (non-colonization), biofilm lifestyle (colonization) and lysis lifestyle (drug release). (c) The bacterial lifestyle transitions were programmed by manipulating the intracellular c-di-GMP level through altering the LPD of the NIR. (d) The level of c-di-GMP increases with LPD and the profile correlated with the expression of *PA2133*. (e) The simulation results show the relationship between the threshold of LPD for biofilm formation and the intensity of the RBS in front of *PA2133*. The threshold of c-di-GMP for biofilm formation was set at 1 μM in the model. When the intracellular c-di-GMP concentration was >1 μM, the bacterial lifestyle changed from planktonic to biofilm. * indicates the experimental results to calibrate the kinetic constants in the model and the color represents the bacterial lifestyle. (f) The RBS in front of *PA2133* was fixed when simulating the effect of the *Q* gene expression level on the critical LPD threshold for bacterial lysis. The dots represent engineered strains with different RBSs in front of *Q*.

By regulating the NIR-responsive c-di-GMP levels, the lifestyle-programmable cancer therapy system can be easily switched between distinct bacterial lifestyles (Fig. 1b). To be specific, under darkness or a Low-LPD of NIR, bacteria are prevented from adhering to the tissue sites; when a Medium-LPD of NIR is supplied, light-activated diguanylate cyclase (BphS) causes an increase in the c-di-GMP level and subsequently enhances biofilm formation; upon illumination with a High-LPD of NIR, lysis is triggered and the pore-forming antitumor toxin Hemolysin E (HlyE) is released to induce tumor necrosis [29,60].

### Modeling of dynamic programming bacterial lifestyle transitions

The genetic circuit for programming bacterial lifestyle transitions consists of four modules involving 22 different species and 30 chemical reactions (Supplementary Tables S4–S6) following mass action kinetics. In order to define an optimal strategy for the subsequent high-throughput construction and screening of engineered bacteria, we developed a simulation model based on a chemical reaction network (CRN) to quantitatively characterize the genetic circuit for bacterial lifestyle programming (Fig. 4a). First, we set the key parameters of the model according to previous studies and performed a parameter calibration in order to represent the real behaviors of each species in the experiment. We optimized the parameters in the photon activation and c-di-GMP signaling network (Supplementary Table S7) to test whether the changes in the model behavior were consistent with the experimental data, based on the analysis results of the effects of PA2133 expression at the threshold level of LPD for promoting bacterial biofilm formation (Fig. 1e). The modeling results indicate that the concentration of c-di-GMP in bacteria increases with LPD, which is consistent with data from the literature [58]. Moreover, we found that increased expression of *PA2133* by replacing promoter J23109 with J23105 inhibits bacterial colonization on surfaces, thus requiring a higher LPD level to promote biofilm formation (Supplementary Table S8). The simulation results with the calibrated parameters (Supplementary Table S7) show that bacterial lifestyle can be dynamically programmed by hierarchical modulation of the LPD of NIR (Fig. 1c). Furthermore, we used this model to investigate how these species change with different experimental parameters of the model (Supplementary Fig. S5). Simulation results indicated that the strength of ribosome binding sites (RBSs) upstream of PA2133 (RBS1) and Q (RBS2) greatly affected the lifestyles of engineered bacteria. Specifically, for RBS1, (i) too weak, planktonic lifestyle is hard to generate; (ii) too strong, LPD of NIR to generate biofilm or lysis lifestyle greatly rises and will limit further clinical transformation. For RBS2, (i) too weak, lysis lifestyle is hard to generate even though High-LPD NIR was supplied; (ii) too strong, the range of LPD of NIR for generating biofilm lifestyle will be rather narrow or even does not exist and treatment with Low-LPD NIR will directly lead to a bacterial lysis lifestyle. By analysing the effect of alterations of each parameter within an appropriate range on the model behavior, we found that the LPD range for maintaining the bacterial lifestyle was jointly determined by the expression levels of *PA2133* and *Q* (Fig. 1f).

### Programming bacterial lifestyle transitions via hierarchically regulating the LPD of NIR

**Figure 2.**
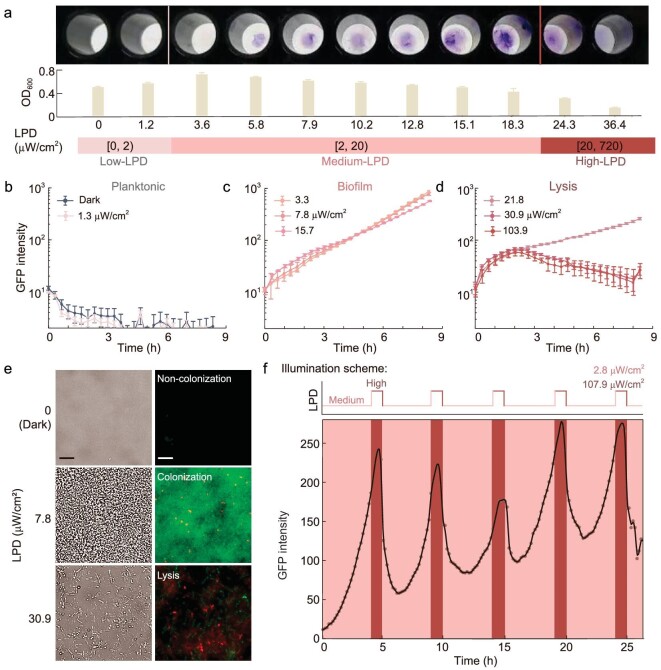
Programming bacterial lifestyles via hierarchically regulating the LPD of NIR. (a) Phenotypic characterization of engineered strains reveals the influence of the LPD of NIR on bacterial lifestyle. Bacteria cultured statically in a 96-well plate were irradiated from the bottom with red light of different LPDs (0–720 ${\rm{\mu W/c}}{{\rm{m}}}^{\rm{2}}$) for 12 h at room temperature. Crystal violet staining results (upper), OD_600_ of the culture supernatant (center) and corresponding LPDs applied (lower). LPD is divided into three intervals (denoted as Low-, Medium- and High-LPD), which were used to manipulate three bacterial lifestyles, respectively (planktonic, biofilm and lysis lifestyles). (b)–(d) Fluorescence profiles of H017 on the surface of microfluidic devices treated with Low-LPD (b), Medium-LPD (c) and High-LPD (d) of NIR. (e) Representative bright field (BF) and fluorescence images of H017 grown for 8 h in the microfluidic device under comparative Low-, Medium- or High-LPD following PI staining. (f) Illumination scheme applied (upper) and the resultant fluorescence profile of H017 on the surface of microfluidic devices (lower). Fluorescence profile showed ‘biofilm–lysis’ oscillations along with the repeated input signal of the ‘Medium–High’ LPD switch. Symbols and lines in (b)–(d) and (f) represent the original and smoothing data, respectively. Scale bars for all images are 10 μm. Error bars represent standard error of mean arising from at least three replicates.

Our first goal was to obtain the strains displaying diverse lifestyles within a reasonable range of LPD through screening. In order to generate a series of strains with different expression levels of *PA2133* and *Q*, we constructed and characterized an RBS library with over a 400-fold dynamic range in *P. aeruginosa* (Supplementary Table S10 and Supplementary Fig. S6). Afterward, a self-made portable 96-well illuminator (Supplementary Fig. S7) was employed to test the influence of LPD of NIR on the lifestyle of the engineered strains in a high-throughput manner. Just as expected, PAO1 does not respond to NIR (Supplementary Fig. S8). The strains capable of biofilm formation and lysis, which are distinctive features of our targeted strains, were identified using crystal violet staining and OD_600_ measurement (Supplementary Fig. S10 and S11). After multiple iterations, we successfully obtained the strain carrying the genetic circuit, wherein the *PA2133* and *Q* genes are controlled by RBS010 and RBS017, respectively. This strain, denoted as H107, exhibited three distinct LPD-dependent lifestyles (Fig. 2a).

To characterize the dynamics of the lifestyle transitions of H017 irradiated with NIR of different LPDs, we monitored the bacterial cell density on the surface of the microfluidic devices using a fluorescence microscope (Supplementary Fig. S12a). We observed that when illuminated with relatively Low-LPD (≤2 ${\rm{\mu W/c}}{{\rm{m}}}^{\rm{2}}$), it was difficult for H017 cells to colonize the surface (Fig. 2b and Supplementary Movie S1). In contrast, under Medium-LPD (2–20 ${\rm{\mu W/c}}{{\rm{m}}}^{\rm{2}}$), H017 rapidly divided *in situ* to form microcolonies and then developed into biofilms in 8 h (Fig. 2c and Supplementary Movie S2). Under High-LPD (≥20 ${\rm{\mu W/c}}{{\rm{m}}}^{\rm{2}}$), lysis was observed (Fig. 2d and Supplementary Movie S3). In addition, H017 is highly efficient in lysis with no visible colony form on an agar plate after 24 h of treatment with High-LPD NIR (Supplementary Fig. S12b). Three lifestyles formed on microfluidics was further verified by using 3D fluorescence imaging of bacterial cells after propidium iodide (PI) staining (Fig. 2e). The fluorescence intensity profiles of H017 treated with High-LPD suggested that the photon-activated bacterial lysis system has a response time of >4 h and that the bacterial lysis behavior is asynchronous and lasts for >10 h. To monitor the cellular levels of Q and c-di-GMP, P*cdrA*-gfp [61], a plasmid-based transcriptional reporter of c-di-GMP, was introduced into our engineered strains. The cellular levels of Q predicted by the computational modeling are consistent with our observations that the reporter fluorescence intensity increased with LPD and decreased after bacterial lysis. The wide distribution of fluorescence intensity within bacteria (Supplementary Fig. S13) indicates that the expression noise of protein Q contributes to asynchronous lysis behavior and that bacteria containing high concentrations of Q lyse first [62].

To achieve the dynamic regulation of lifestyle transitions of H017 as predicted by the model, we optimized the illumination scheme in specific experiments. To begin with, 4-h NIR with Medium-LPD (2.75 ${\rm{\mu W/c}}{{\rm{m}}}^{\rm{2}}$) is utilized to promote biofilm formation; then 1-h NIR with High-LPD (107.91 ${\rm{\mu W/c}}{{\rm{m}}}^{\rm{2}}$) is used to induce bacterial lysis. The entire lysis process takes ∼5 h. Bacterial intracellular c-di-GMP levels differ from each other under the same illumination conditions due to the noise in gene expression and bacteria that reached the c-di-GMP threshold for lysis lysed first (Supplementary Fig. S13f). With a shorter period of NIR illumination with High-LPD, some bacteria will not undergo the lysis process and regenerate biofilm after another treatment with Medium-LPD NIR (Supplementary Fig. S14b). The percentage of lysis-able bacteria in each biofilm–lysis lifestyle transition was ∼60% based on the reduction in fluorescence intensity (Supplementary Fig. S14c). This ‘biofilm–lysis’ lifestyle transition can be repeated cyclically under periodic NIR illumination of 4-h Medium-LPD and 1-h High-LPD (Fig. 2f and Supplementary Movie S4). The results above suggest that the LPD of NIR employed to program the bacterial lifestyle transitions is tunable within the range of the ‘${\rm{\mu W/c}}{{\rm{m}}}^{\rm{2}}$’ level, which would greatly increase the availability of clinical treatments for deep-seated tumors.

### Programming bacterial lifestyle transitions facilitates the controllable release of therapeutic agents

**Figure 3.**
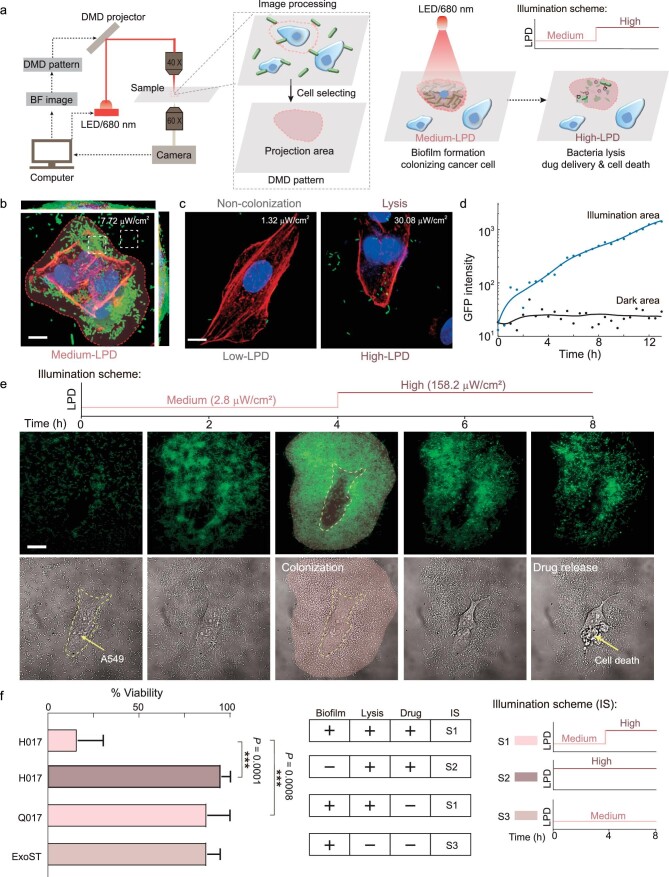
Programming ‘biofilm–lysis’ lifestyle transitions facilitates controllable release of therapeutic molecules. (a) Schematic of the experimental workflow of programming bacterial lifestyles on the surface of targeted human lung cancer cells A549 using a microscope. The bright field (BF) images of the cancer cells on microfluidics were acquired by using a ×60 oil objective and then the profiles of selected cells were projected onto the cover glass by using a digital micro-mirror device-based (DMD) light-emitting diode (LED) projector through a ×40 air objective. The intensity of the LED was controlled by using MATLAB in real time. Schematic diagram of programming the ‘biofilm–lysis’ lifestyle transition in the targeted area by applying an illumination scheme. (b) Confocal projections of biofilms formed by RecA-H036 (labeled with green fluorescent protein) co-cultured with fixed A549 cells under Medium-LPD of NIR (680 nm) for 8 h, and then stained with phalloidin (FITC, red) and DAPI (blue). (c) Laser scanning confocal micrographs of RecA-H036 co-cultured with fixed A549 cells under comparative Low- and High-LPD of NIR. Images are representative of the NIR light area of the bacterial lifestyles monitored throughout the duration of the experiment, including three lifestyles: no attachment of cells to A549 cells, biofilms formation on A549 cells and bacterial lysis. The applied LPDs are shown in the upper right of the picture. Scale bar, 10 μm. (d) Fluorescence profiles of bacteria in the illumination area or dark area as depicted in (b) (white dashed squares). (e) Schematic diagram of illumination scheme applied (upper) and the resultant time course of BF and fluorescence images of H017 co-cultured with A549 live cancer cells for 8 h in the microfluidic device, sequentially visualizing H017 biofilm formation, lysis and A549 cancer cell death. Scale bar, 20 μm. (f) Percentage viability of A549 live cells. A549 live cells co-cultured with H017 and treated with illumination scheme S1 to promote biofilm formation, bacterial lysis and drug release; co-cultured with H017 and treated with S2 to generate direct bacterial lysis and drug release; co-cultured with Q017 and treated with S1 to promote biofilm formation and bacterial lysis; co-cultured with ExoST and treated with S3 to promote biofilm formation. ***P < 0.001, unpaired two-tailed *t*-test. Error bars represent SEM arising from at least three replicates.

To validate the role of the three bacterial lifestyles in tumor therapy *in vitro*, we co-cultured fixed human lung cancer cells A549 with our engineered bacteria in a microfluidic device (Supplementary Fig. S15) and irradiated specific cancer cells with NIR of different LPDs. The illumination area can be determined according to the contour of the A549 cells with an established protocol (Fig. 3a). We observed that A549 cells were completely covered with biofilms that could resist the flow-induced shear under NIR irradiation with Medium-LPD (7.72 ${\rm{\mu W/c}}{{\rm{m}}}^{\rm{2}}$) (Fig. 3b), whereas in the absence of NIR illumination, bacteria cannot colonize the surface of A549 cells (Fig. 3c). While High-LPD NIR was supplied, bacterial lysis was triggered (Supplementary Fig. S16). These results suggest that the formation of biofilm facilitates the strong bacterial adhesion of the bacteria on the surface of the A549 cell. Thus, we speculated that biofilm formation in tumor sites may promote colonization. In addition, the planktonic lifestyle of therapeutic strains may contribute to the non-colonization of bacteria on normal tissues and decreased systemic cytotoxicity.

To verify the benefits of bacterial lifestyle transitions in tumor therapy, we monitored the density of the bacteria in the illumination area by using a fluorescence microscope (Fig. 3d) and recorded the survival ratio of cancer cells co-cultured with engineered bacteria using different illumination schemes (Fig. 3e). We found that the density of the bacteria in the illumination area is proportional to the duration of NIR illumination with Medium-LPD (Supplementary Fig. S15e). Thus, we hypothesized that programming the process of the bacterial lifestyle (biofilm–lysis) transition could enable the controllable release of therapeutic agents into the TME. To test this hypothesis, an illumination scheme (Fig. 3b) is adopted wherein NIR with Medium-LPD (2.75 ${\rm{\mu W/c}}{{\rm{m}}}^{\rm{2}}$) was applied for the first 4 h and then LPD was raised to 158.22 ${\rm{\mu W/c}}{{\rm{m}}}^{\rm{2}}$ for the next 6 h. We extended the treatment time of High-LPD NIR in order to increase the proportion of lysis-able bacteria in the biofilm for adequate drug release. We observed that the survival ratio of A549 cells co-cultured with H017 was <20% (Fig. 3b and Supplementary Movie S5) and there was no significant difference in the viability and morphology of A549 cells without NIR illumination. In this study, cell death can be determined by morphological changes and verified by using PI staining (Supplementary Fig. S17). Nevertheless, cell viability is slightly affected by direct lysis of H017 without biofilm formation (Fig. 3c and Supplementary Movie S6), lysis of Q017 after biofilm formation without drug release (Supplementary Fig. S18) and biofilm formation of ExoST without drug release (Supplementary Movie S7). These results suggest that the localization, timing and dosage of therapeutic agents in the process of BMCT can be precisely manipulated according to our needs by programming bacterial lifestyle transition.

### Programming bacterial lifestyle transitions in solid tumors by NIR

**Figure 4.**
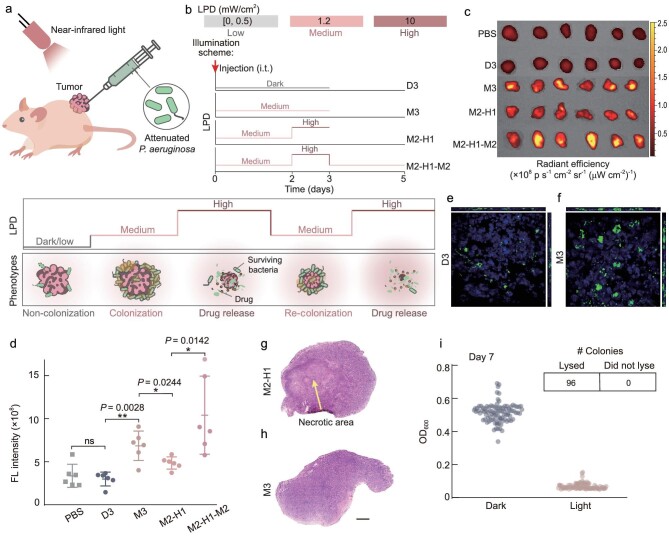
Programming bacterial lifestyles in solid tumors by NIR light. (a) Schematic diagram of the dynamic programmable manipulation of intratumoral bacterial lifestyles for cancer therapy. Six to 8-week-old female BALB/c nude mice (*n* = 6 per group) were subcutaneously (s.c.) inoculated with 10^7^ A549 cells into the flank. When the tumor volumes reached ∼100 mm^3^, mice were divided into five treatment groups. Group PBS received intratumoral (i.t.) injection with PBS and were cultured normally. The remaining four groups received i.t. injection with 5 × 10^7^ colony forming units (CFUs) of H017 and then treated with various illumination schemes as follows: group D3 was kept in dark conditions for 3 days; group M3 was illuminated with Medium-LPD for 3 days; group M2-H1 was illuminated with Medium-LPD for 2 days and High-LPD for 1 day successively; group M2-H1-M2 was treated with M2-H1 first and then illuminated with Medium-LPD for another 2 days. (b) Schematic diagram of experimental illumination schemes and specific values of each LPD. The i.t. injection time point is depicted by a red arrow. (c) IVIS images of tumors after treatment. (d) Fluorescence intensity of intratumoral bacteria determined by using IVIS. Each dot in the plot diagram represents one counted tumor. Representative confocal microscopy image of frozen tumor sections (15 μm thick) taken from mice in group (e) D3 and (f) M3. DAPI (blue), H017 (green). H&E stained sections (8 μm thick) of tumor tissues taken from mice in group (g) M2-H1 and (h) group M3. (i) Distribution of OD_600_ values of bacterial supernatant originating from 96 single colonies cultured under darkness or a duplicate cultured with High-LPD illumination for 6 h. The colonies were harvested from tumors after treatment under Medium-LPD red light for 7 days. The grid above shows the number of successful lysis events. **P < 0.01; *P < 0.05; ns, not significant, unpaired two-tailed *t*-test. Error bars represent SEM arising from at least three replicates.

We employed a subcutaneous A549 tumor model to investigate whether the lifestyle of H017 *in vivo* can be programmed by modulating the LPD of NIR as a proof-of-concept. We irradiated A549 tumor-bearing mice according to various NIR illumination schemes after intratumoral injection of H017. We first recalibrated the specific values for NIR with Medium-LPD and High-LPD, respectively (Fig. 4b), based on the tissue penetration efficiency of NIR [63]. The intratumoral bacteria was visualized by using an In Vivo Imaging System (IVIS). The fluorescence intensity of the tumor tissues injected with H017 under dark conditions for 3 days (D3) was indistinguishable from that of phosphate buffer saline (PBS)-injected controls (Fig. 4c and d, *P* = 0.5737) and showed a significant decrease in bacterial quantities compared with tumors obtained at 4 h post-intratumoral injection (Supplementary Fig. S19, *P* = 0.0144), suggesting that H017 could not colonize tumor tissues without NIR illumination. Tumors under NIR illumination with Medium-LPD (1.2 ${\rm{mW/c}}{{\rm{m}}}^{\rm{2}}$) for 3 days (M3) exhibited a higher bacterial fluorescence intensity than that of D3 (Fig. 4c and d, *P* = 0.0028), indicating a higher density of bacterial colonization. Besides, the confocal fluorescence images of sections of tumors treated with M3 revealed that biofilms were formed in tumors (Fig. 4e and f).

Next, we sought to assess whether the lifestyle transitions of intratumoral bacteria could be dynamically programmed by adjusting the NIR illumination scheme *in vivo* over a few days. We found that the fluorescence intensity of tumors treated with M2-H1 was lower than that of tumors treated with M3. Moreover, the tumor treated with M2-H1 and stained with hematoxylin and eosin displayed cellular necrosis (Fig. 4g and Supplementary Fig. S20), whereas tumors treated with M3 maintained vitality (Fig. 4h). The release of pore-forming cytolysin HlyE can lead to tumor necrosis [60]. Thus, we speculate that lysis of bacteria within biofilm in the tumors after treatment with M2-H1 leads to sufficient HlyE release (Supplementary Fig. S20). To verify whether the remaining bacteria after High-LPD illumination can grow and form biofilm again, we compared the fluorescence intensity of tumors treated with M2-H1-M2 and M2-H1 (Fig. 4d, *P* = 0.0142). The results showed that biofilm gradually reformed under another 2-day Medium-LPD of NIR illumination. Moreover, the intratumoral bacterial lifestyle transitions can be programmed with quite Low-LPD of NIR compared with that used in PTT, making it promising for further clinical application for safety concerns. To confirm the long-term stability of our designed inducible lysis module after bacterial colonization in tumors, the growth of the bacterial colonies extracted from within tumors under 7-day NIR illumination with Medium-LPD (1.2 ${\rm{mW/c}}{{\rm{m}}}^{\rm{2}}$) was tracked under darkness and High-LPD of NIR illumination, respectively. We observed that colonies all lysed under NIR illumination with High-LPD (Fig. 4g), indicating that NIR sensitivity and lysis capacity were maintained *in vivo*.

### Programming the intratumoral bacterial lifestyle transitions enhances therapeutic efficacy

Given the ability to release antitumor drug HlyE in tumor grafts using NIR, we utilized H017 as a delivery vector to inhibit tumor growth (Supplementary Fig. S21). Under 20-day NIR illumination with High-LPD, the tumor damage in the A549 tumor-bearing mice was determined by using HE and TUNEL staining assays (Supplementary Fig. S22). The mass of tumors with multiple injections of H017 under NIR illumination with High-LPD was significantly smaller than that of the control group (under darkness). In addition, the growth rate of tumors injected with ExoST under NIR illumination with High-LPD was indistinguishable from that observed under darkness, suggesting that the effect of biofilm formation and NIR illumination on tumor growth is negligible (Supplementary Fig. S23). These results show that H017 is a highly efficient drug delivery system that can inhibit the growth of subcutaneous solid tumors *in vivo*. Though multiple injections of H017 followed by NIR illumination with High-LPD can inhibit tumor growth, excessive injections are not convenient and are prone to side effects.

**Figure 5.**
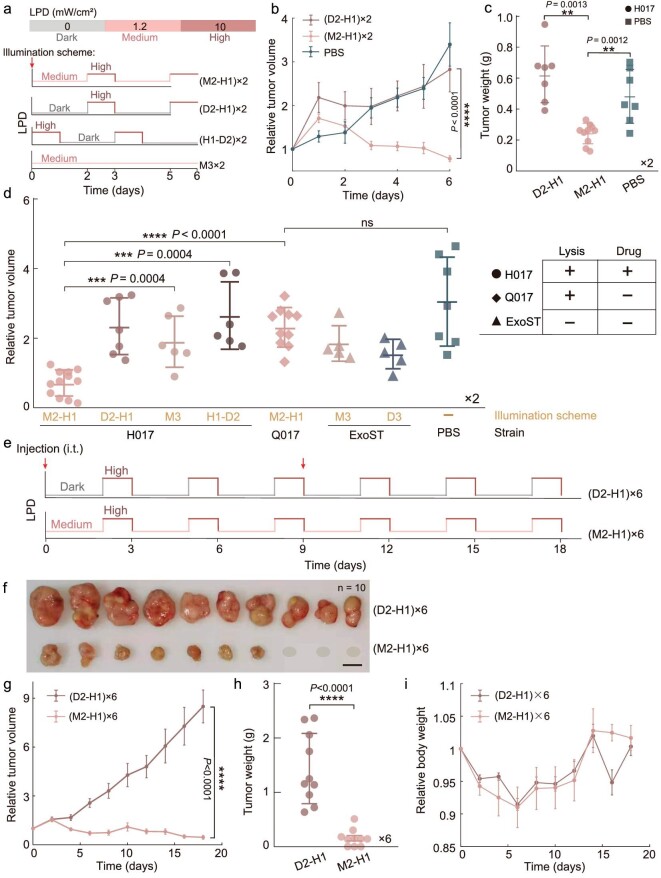
Tumor regression in mice induced by programming intratumoral bacterial lifestyles. Six to 8-week-old female BALB/c mice (*n* = 5–12) were subcutaneously (s.c.) inoculated with 10^7^ A549-mCherry cells into the flank. When the tumor volumes reached ∼100 mm^3^, experiments were conducted. (a) Schematic diagram of the experimental illumination scheme. All abbreviations have been mentioned except for D2-H1 and H1-D2. D2-H1 means culturing in dark conditions for 2 days and illuminating with High-LPD for 1 day; H1-D2 means illuminating with High-LPD for 1 day and then culturing in dark conditions for 2 days; ×2 means the process was repeated for two cycles. Time points of intratumoral (i.t.) injection of 5 × 10^7^ colony forming units (CFUs) of engineered bacteria (H017, Q017, ExoST) or PBS are depicted in red arrows. (b) Relative tumor volume over time and (c) distribution of weight of tumors. A549-mCherry tumor-bearing mice that received i.t. injection of H017 were treated with illumination scheme (D2-H1) ×2 or (M2-H1) ×2; mice treated with i.t. injection of PBS and incubated normally were used as a control. (d) Distribution of relative tumor volumes. A549-tumor-bearing mice that received i.t. injection of H017 and treated with (M2-H1) ×2, (D2-H1) ×2, M3 ×2, (H1-D2) ×2 or i.t. injection of Q017 and treated with (M2-H1) ×2; or i.t. injection of ExoST and treated with M3 ×2, D3 ×2 or i.t. injection of PBS and incubated normally. Functional compositions of each engineered strain are also depicted in the right-hand picture. (e) Schematic diagram of the illumination schemes applied; ×6 means the process was repeated for six cycles. Time points of A549-mCherry tumor-bearing mice that received i.t. injection of 5 × 10^7^ CFUs of H017 are depicted by red arrows and (f) images of the resultant tumors (scale bar, 1 cm). The gray ellipse indicates that the tumor has been completely eliminated. (g) Relative tumor volume and (h) distribution of weight of tumors. (i) Relative body weight of A549-mCherry tumor-bearing mice over time. ****P < 0.0001; ***P < 0.001; **P < 0.01; ns, not significant, unpaired two-tailed *t*-test. Each dot in the plot diagram represents one counted tumor. Error bars represent SEM arising from at least three replicates.

To investigate whether optimizing the process of bacterial lifestyle transitions enhances therapeutic efficacy, we monitored the relative tumor volume variation of the tumor treated with a single intratumoral injection of H017 and then illuminated the tumor using different illumination schemes as shown in Fig. 5a. Compared with other illumination schemes, tumor activity in mice injected with H017 using the illumination scheme (M2-H1) ×2 was significantly reduced over a period of 6 days (Fig. 5b–d and Supplementary Fig. S24). Moreover, there was no significant difference in the tumor volume between A549-bearing mice injected with Q017 using the illumination scheme (M2-H1) ×2 and those treated with ExoST using the illumination scheme M3 ×2 or D3 ×2 (Fig. 5d), indicating that neither natural cellular contents of our chassis bacteria nor biofilm formation of our chassis bacteria had a therapeutic effect on solid tumors.

Considering that our designed ‘biofilm–lysis’ transition can promote bacteria recolonization after lysis, sustained and controlled drug release is possible if an appropriate illumination scheme is adopted. To improve the therapeutic efficacy, we extended the illumination scheme to three cycles and repeated the intratumoral injection–illumination treatment (Fig. 5e). We have demonstrated that the generation of the biofilm lifestyle of H017 after treatment with Medium-LPD NIR contributes to the increased colonization densities while H017 with a planktonic lifestyle after treatment with dark conditions was hard to colonize in solid tumors (Supplementary Fig. S19). Meanwhile, H017 showed significantly increased bacterial colonization densities after treatment with M2-H1 compared with treatment with D2-H1 (Supplementary Fig. S19, *P* < 0.0001). Hence, we think that treating H017 with Medium-LPD NIR first and then High-LPD NIR would lead to more drug release and better therapeutic efficacy. We found that 30% of the tumors disappeared after treatment and the growth of the remaining tumors was significantly inhibited with double injections and multiple cycles of NIR illumination (Fig. 5f–h). It should be noted that there were no significant adverse effects observed according to changes in relative body weight throughout the observation period (Fig. 5i). In addition, translocation of bacteria in lung and liver was observed in only 20% of the mice. While tumor regression occurred, there were no bacteria detected in main tissues and we speculated that they were cleared by the immune system (Supplementary Fig. S25). Overall, our *in vivo* experiments indicated that complete tumor eradication can be achieved by programming bacterial lifestyle transitions with an optimized illumination scheme.

## DISCUSSION

We have developed a programmable bacterial lifestyle transitions system based on NIR light for the controllable release of antitumor drugs into the TME and exemplify a methodology for promoting bacterial colonization of tumor sites. Clinical studies have shown that increasing tumor-specific colonization by bacteria can greatly enhance therapeutic efficacy and reduce toxicity to normal tissues. In this study, the formation of biofilm in tumor tissue significantly enhanced bacterial colonization. Large amounts of bacteria were observed to persist within tumors for ≥7 days (Fig. 4c), thereby releasing more therapeutic agents for better therapeutic efficacy (Fig. 5c). The synthesis of EPS during the process of biofilm formation forms sticky and tangled fibers that connect bacteria cells to each other and to various surfaces [64]. Thus, we speculate that the biofilm lifestyle acts as a defense barrier that protects the bacteria embedded in the EPS matrix against innate host defense [65] and shear caused by fluid flow [66]. In addition, EPS produced on the surface of cancer cells inhibits the adhesion of cancer cells to endothelial cells, resulting in metastasis disruption [45,67]. Hence, the introduction of a biofilm lifestyle into our engineered bacteria may bring much more unexpected antitumor effects. While the hypothesis above requires further validation, our results indicated that biofilms have shown great application potential in BMCT. Apart from programming the intratumoral bacterial lifestyle, we also engineered the extratumoral bacterial lifestyle, which is uncommon and difficult to achieve in the current BMCT. The therapeutic strains in our system, which continuously express PA2133, produce a relatively low level of c-di-GMP intracellularly and cannot colonize various tissue surfaces under darkness (Fig. 3a). This is also confirmed by the result that the fluorescence intensity of tumors injected with H017 under darkness for 3 days was essentially indistinguishable from that of tumors injected with PBS (Fig. 4c). The induction of PA2133 (c-di-GMP hydrolysis module) results in the maintenance of a planktonic lifestyle that exhibits high susceptibility to antimicrobials [68], allowing *P. aeruginosa* biofilms to disperse and thus be eliminated by the host immune system [69]. The design of a planktonic lifestyle together with the lysis lifestyle greatly improved the biosafety of our engineered strain. Collectively, our results indicate that a bacterial lifestyle can be easily switched between two or more lifestyles by modulating the NIR illumination scheme and programming lifestyle transitions of the engineered bacteria according to various environments would greatly improve safety while enhancing the antitumor efficacy. Through our designed genetic circuit, bacteria can achieve multiple cycles of ‘biofilm–lysis’ transition, resulting in sustained release of drugs to eliminate tumors.

Although we preliminarily verified the therapeutic efficacy of attenuated *P. aeruginosa* via optogenetic manipulation of the intratumoral bacterial lifestyle transition in a subcutaneous mouse model, we acknowledge that there is still much work that remains to be done for preclinical application, including genetic stability improvement, antitumor agents replacement, biosafety improvement, development of a specialized device for optogenetic manipulation and validation of the therapeutic efficacy of H017 or other derivative *P. aeruginosa* strains on an orthotopic lung cancer model. We observed that the therapeutic efficacy gradually diminished after three cycles of the illumination scheme M2-H1 *in vivo*. Some tumors disappear completely with a second injection of the engineered bacteria (Fig. 5f). The possible reasons for the above results include: (i) the loss of the plasmid in the bacteria containing the lysis trigger gene *Q* and the mutation of bacteria leads to the failure of the lysis system; (ii) due to the limitations of experimental conditions, we were unable to obtain the best illumination scheme by observing the bacterial density within the tumor *in situ*. Further improvements would be derived from strategies for longer-term circuit stability and the utilization of additional therapeutic agents. Another issue that needs to be considered is that *P. aeruginosa* are bacteria with a high level of horizontal gene transfer (HGT). In order to stop HGT from the engineered strain to commensal *P. aeruginosa* strains in the lung or other commensal bacteria, two strategies would be useful: (i) abolish the use of plasmid to reduce mobile genetic elements and integrate all functional modules into the *P. aeruginosa* chromosome to remove antibiotic resistance genes and enhance genetic stability [8]; (ii) delete T4SS pilus-related genes [70] to avoid conjugative gene transfer [71].

We employed NIR as a trigger for hierarchically manipulating complex bacterial lifestyle transition via an optogenetic tool BphS and this manipulation is hard to achieve through chemical inducers or specific TME signals, opening up the possibility of customized cancer therapy. Optogenetics provides a promising approach for gene- and cell-based therapies via precisely manipulating various cellular activities with high spatio-temporal resolution [72]. And the deep tissue penetration capacity of NIR light offers a non-invasive treatment modality for a variety of diseases [73,74]. For the practical application of manipulating bacteria behaviors in an orthotopic lung cancer model, a custom-designed device could be adopted. The development of advanced functional materials makes more and more high-performance, light and smart wearable materials available nowadays; among them, bioelectronic implants have already been adopted in medical application [75]. To control the enzymatic activity of BphS *in vivo*, which was also adopted in our genetic circuit design, Shao *et al.* developed an implanted Hydrogel light-emitting diode (LED) device that can be remotely controlled by a smartphone [76]. Besides, the illumination intensity and pattern of multiple light spots, especially lasers, can be easily controlled by computer algorithms, enabling neuroscientists to stimulate hundreds of precisely targeted neurons simultaneously in two-photon holographic optogenetics [77]. We think these are alternatives that could also be used for regulating bacterial behaviors in the practical application of an orthotopic lung cancer model after careful modification according to our experiment requirements.

As a newly developed chassis strain, there is no doubt that people have raised extreme concerns about the biosafety of engineered *P. aeruginosa.* In fact, model strains in BMCT like *Salmonella, E. coli* and *L. monocytogenes* are all human pathogens, but they have shown great feasibility and antitumor effects in BMCT by attenuation or by constraining their growth in normal tissues through genetic modification. We found that there was some probability of bacterial translocation to normal tissues (Supplementary Fig. S25). Although there was no significant damage according to pathological observation of the main organs 7 days post-s.c. injection of H017 (Supplementary Fig. S1g), it is critical to strictly limit bacterial growth inside solid tumors for biosafety concerns. Construction of an auxotrophic mutant should be employed in future studies (Supplementary Table S12). Intravenous administration is widely adopted in BMCT and bacteria are transported systemically in this manner. Even though they would be eventually eliminated by the host immune system and specially colonize solid tumors, they are likely to cause unexpected damage since they are directly transported to normal tissues first. To make the system more clinically relevant, other routes of therapeutic administration need to be considered. Considering *P. aeruginosa* is a strain with the capacity to typically colonize lung tissue, intranasal administration could be adopted in clinical application to directly deliver engineered bacteria to the tumor location without disturbing normal tissues. In addition, the mutant *P. aeruginosa* lacking *exoS* and *exoT* was hard to spread systemically when intranasally administered [54], which may also improve the biosafety of the engineered *P. aeruginosa* strain. Although these strategies require further verification, it will not be hard to achieve a safer engineered *P. aeruginosa* strain for preclinical study with the booming synthetic biology.

## MATERIALS AND METHODS

### Construction of plasmids and PAO1 derivatives

To generate an attenuated strain of *P. aeruginosa*, the following virulence factor genes were sequentially deleted from the chromosome of the wild-type strain PAO1: *vfR, exoS, exoT*. The core NIR light-responsive module *bphS* was integrated into the chromosomal *attB* site by using plasmids miniCTX2. The c-di-GMP hydrolysis module *PA2133* and lysis cassette *LKD* was cloned into vector miniTn7 and then inserted into the genome *attTn7* site. Anticancer drug synthesis gene *hlyE* and antiterminator *Q* for activation of the lysis system in response to the c-di-GMP concentration was cloned into vector pUCP20. We also constructed and characterized the RBS library in PAO1 cells. All plasmids were constructed using basic molecular cloning techniques and Gibson assembly. Additional details for construction of the optogenetic and reporter strains are given in the Supplementary text.

### Computational model

Using a CRN representation, we describe and quantify the dynamics of the designed genetic circuit composed of 22 different species (Supplementary Table S6) and 30 chemical reactions (R1–R30 in Supplementary Table S4) that follow the mass action kinetics. A diagram of the model is shown in Supplementary Fig. S4 and the simulation was computationally implemented using a MATLAB toolbox (Simbiology). The parameters of the reactions in this model, obtained from previous literature reports or our experimental results, are listed in Supplementary Table S7. The detailed computational model derivation is described in the Supplementary text.

### Mouse experiments

This study was approved by the Local Ethics Committee for Animal Care and Use (permit number: USTCACUC 1901026) and the experiments involving animals were used according to the animal care regulations of the University of Science and Technology of China. Sacrificed animals were euthanized by cervical dislocation when the tumor size reached 2 cm in diameter or after recommendation by the veterinary staff. Animal experiments were performed on BALB/c nude mice (Charles River) that were allowed to acclimatize to the institutional animal facility for 1 week prior to experiments.

### Statistical analysis

All qualitative images presented are representative of at least three independent duplicate experiments. Mice were randomized into different groups before experiments. All experiments data are expressed as the mean ± standard error of mean. Statistical significance between the control and treated groups was evaluated by using a two-tailed Student's *t*-test using the statistical program of GraphPad Prism software. Differences between experimental groups were considered significant when *P*-values were <0.05. Statistical details of the experiments are included in the figure legends.

## Supplementary Material

nwad031_Supplemental_Files
