# Supplementary figures and images for: Programming the lifestyles of engineered bacteria for cancer therapy
Source: Natl Sci Rev. 2023 Feb 14;10(5):nwad031. doi: 10.1093/nsr/nwad031 (PMC10089584; doi:10.1093/nsr/nwad031)

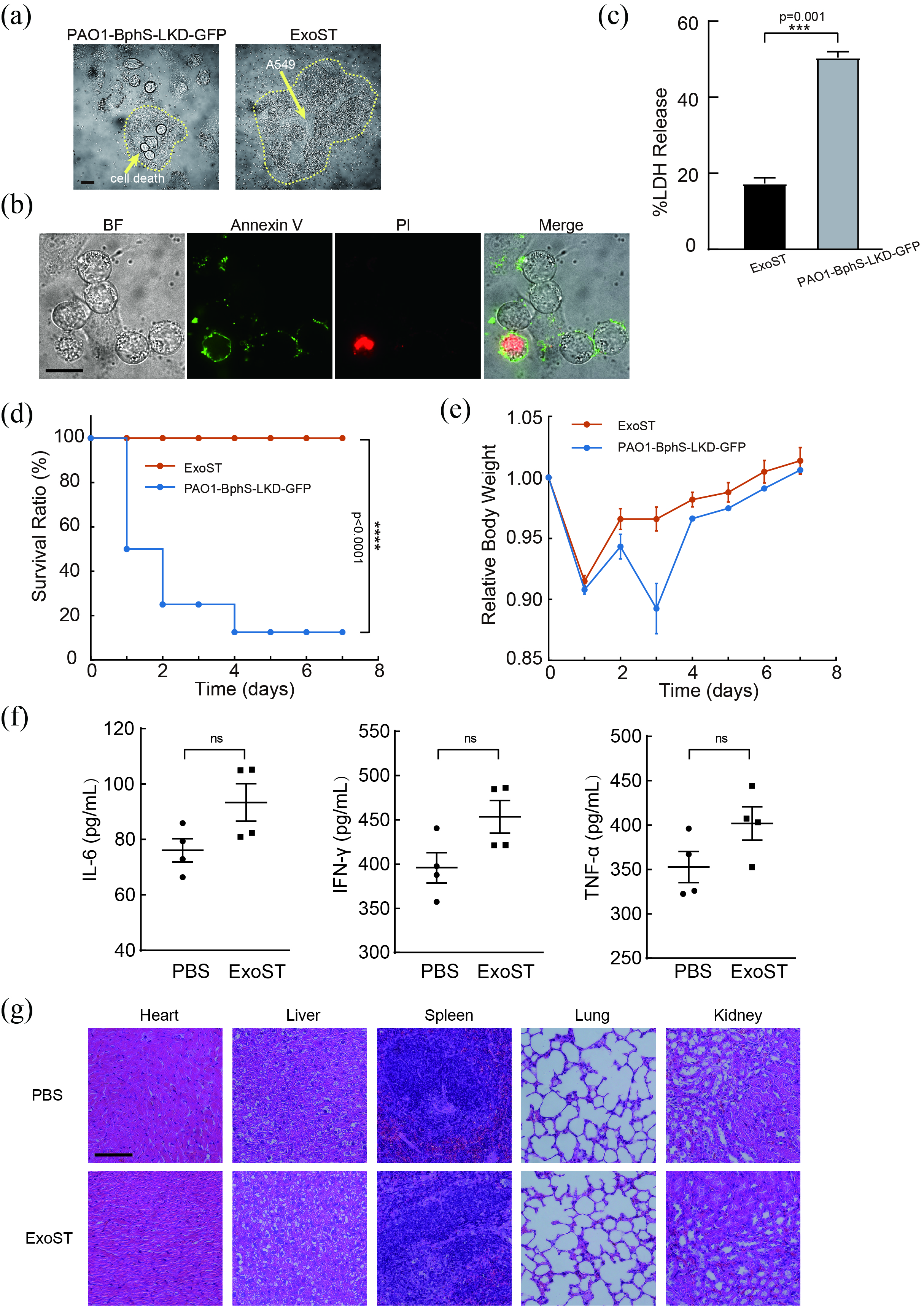

Supplement: nwad031_Supplemental_Files [file nwad031_supplemental_files.zip › supplementary figures/Fig S1.jpg]

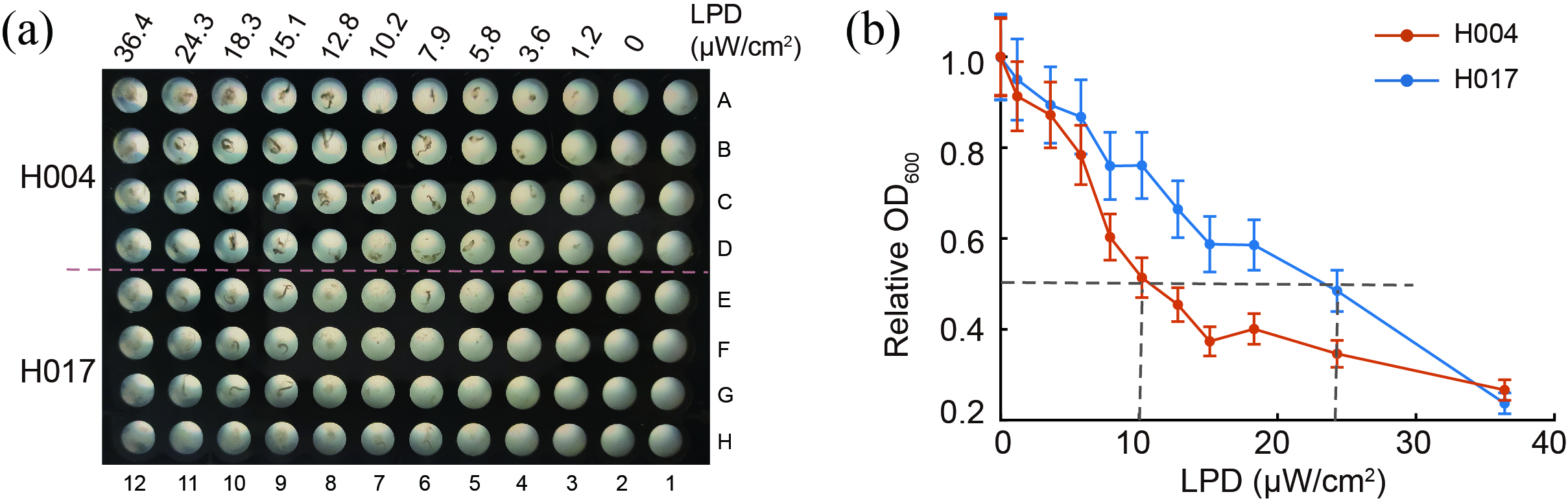

Supplement: nwad031_Supplemental_Files [file nwad031_supplemental_files.zip › supplementary figures/Fig S10.jpg]

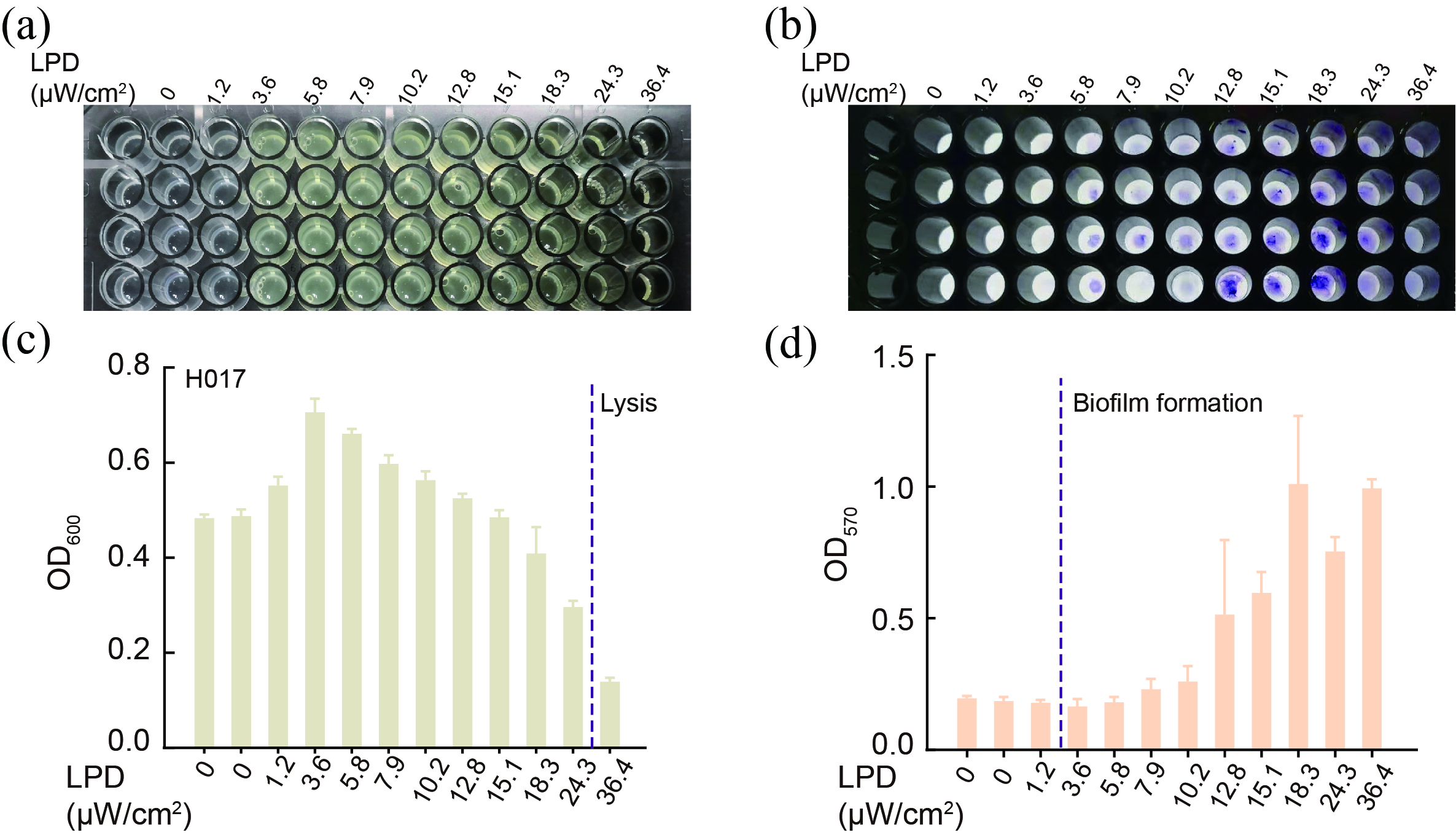

Supplement: nwad031_Supplemental_Files [file nwad031_supplemental_files.zip › supplementary figures/Fig S11.jpg]

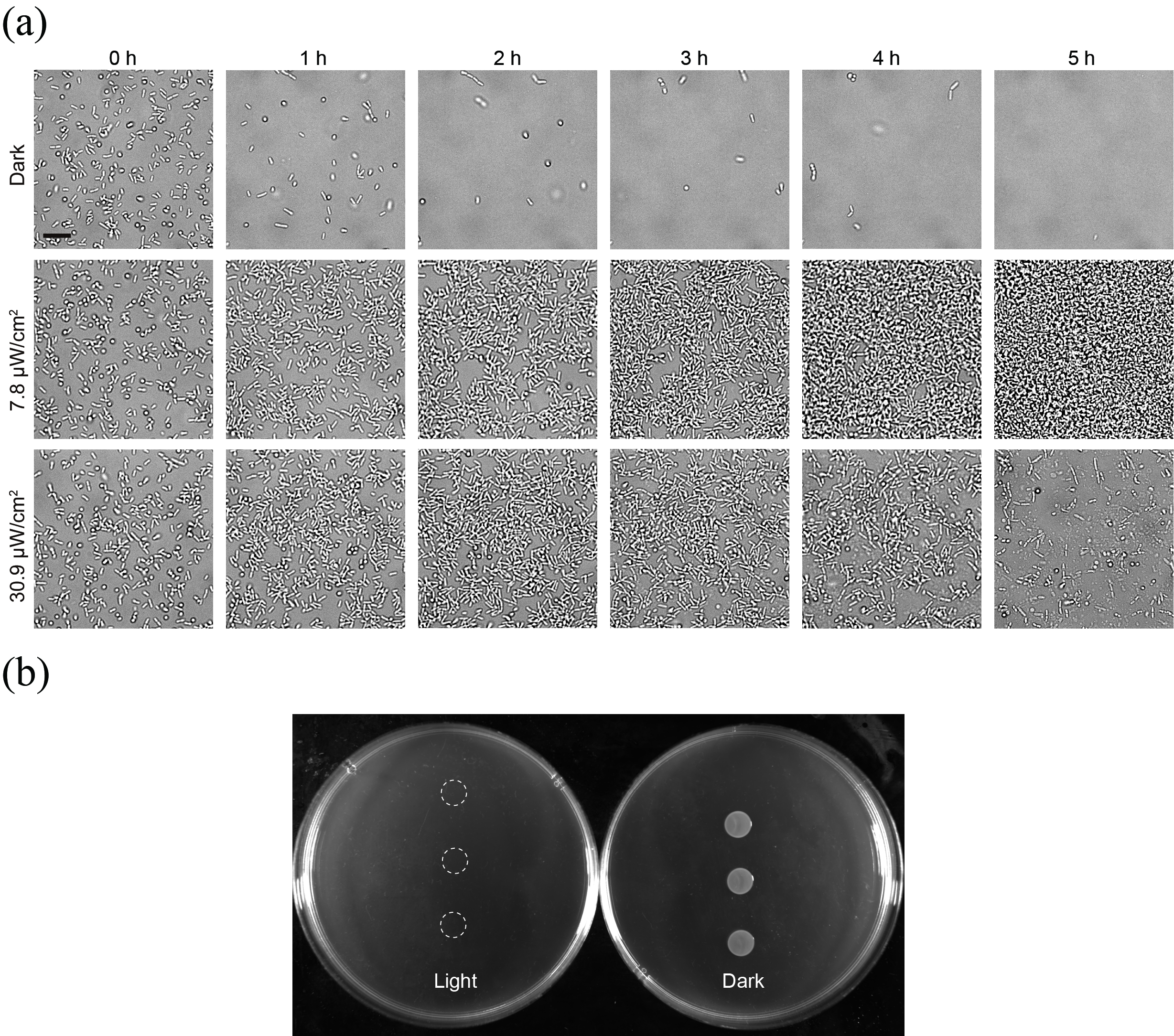

Supplement: nwad031_Supplemental_Files [file nwad031_supplemental_files.zip › supplementary figures/Fig S12.jpg]

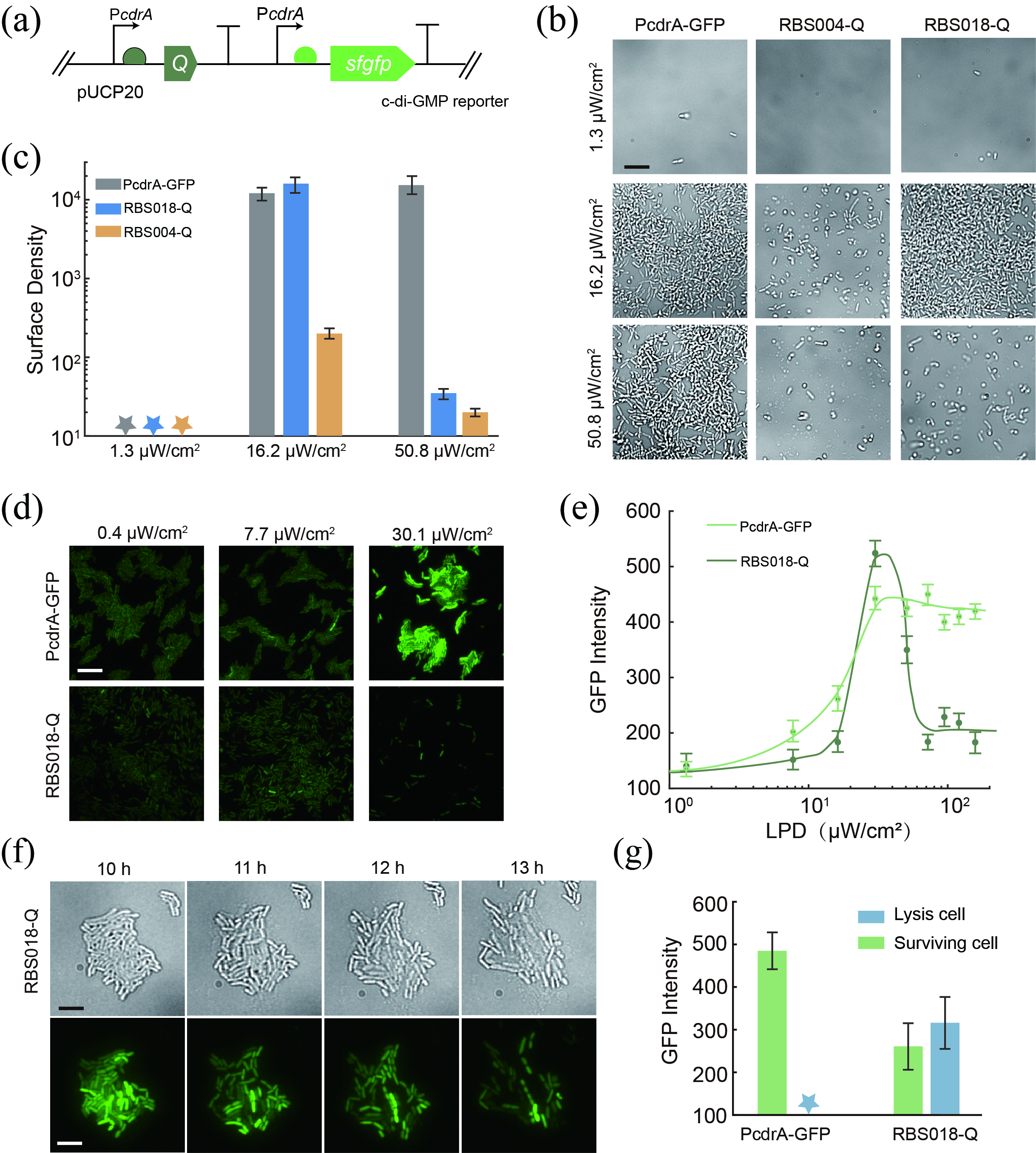

Supplement: nwad031_Supplemental_Files [file nwad031_supplemental_files.zip › supplementary figures/Fig S13.jpg]

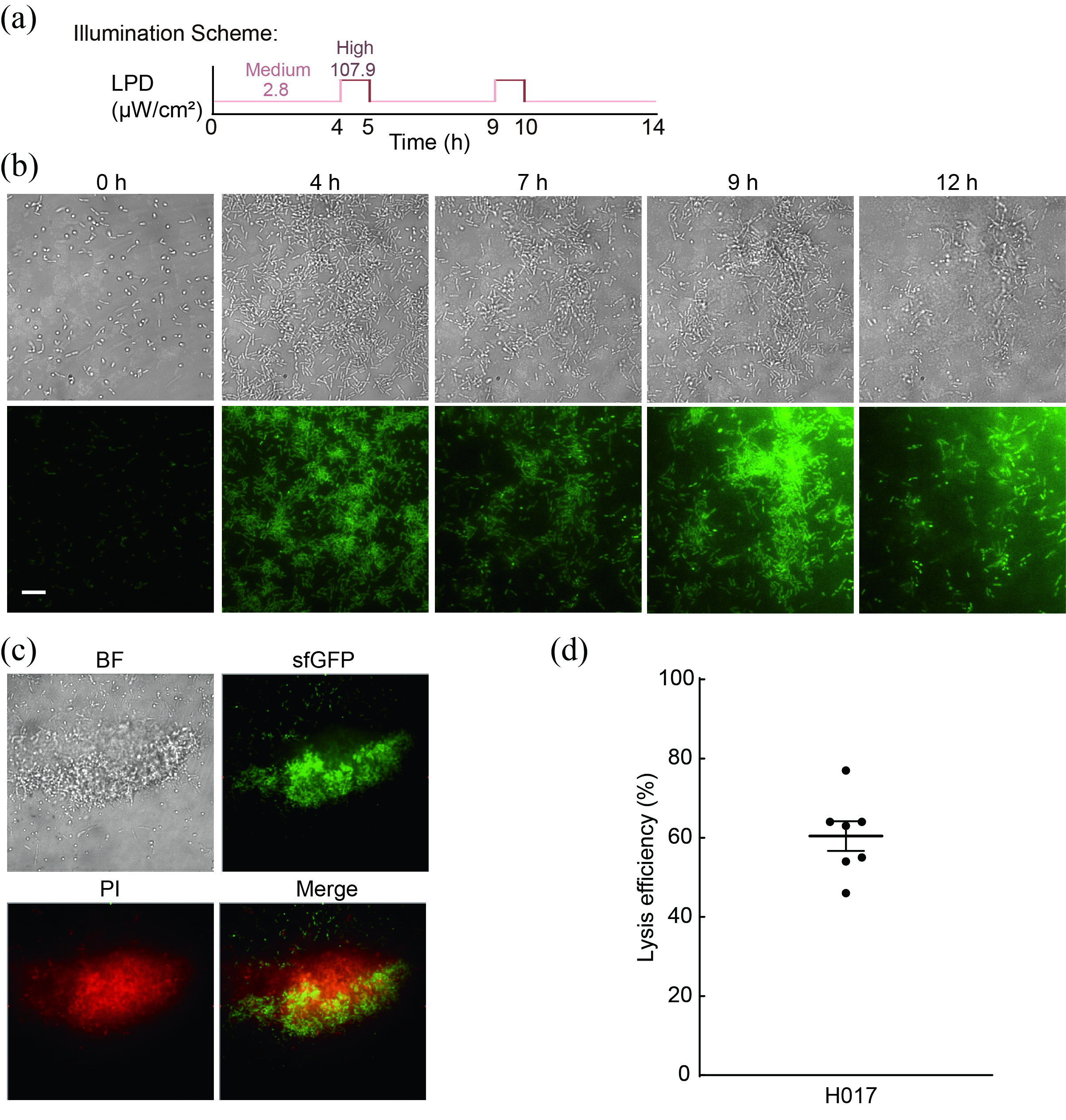

Supplement: nwad031_Supplemental_Files [file nwad031_supplemental_files.zip › supplementary figures/Fig S14.jpg]

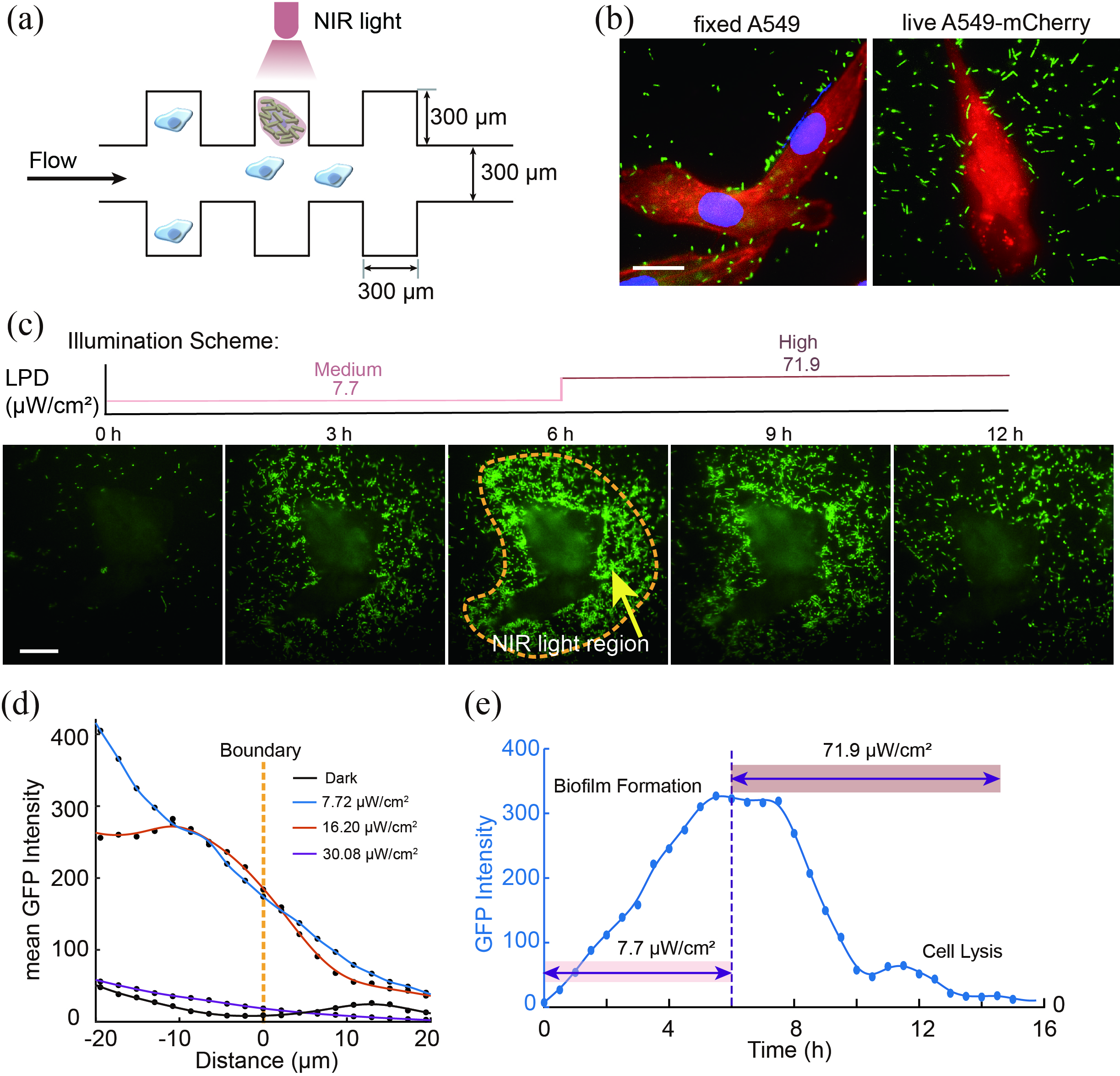

Supplement: nwad031_Supplemental_Files [file nwad031_supplemental_files.zip › supplementary figures/Fig S15.jpg]

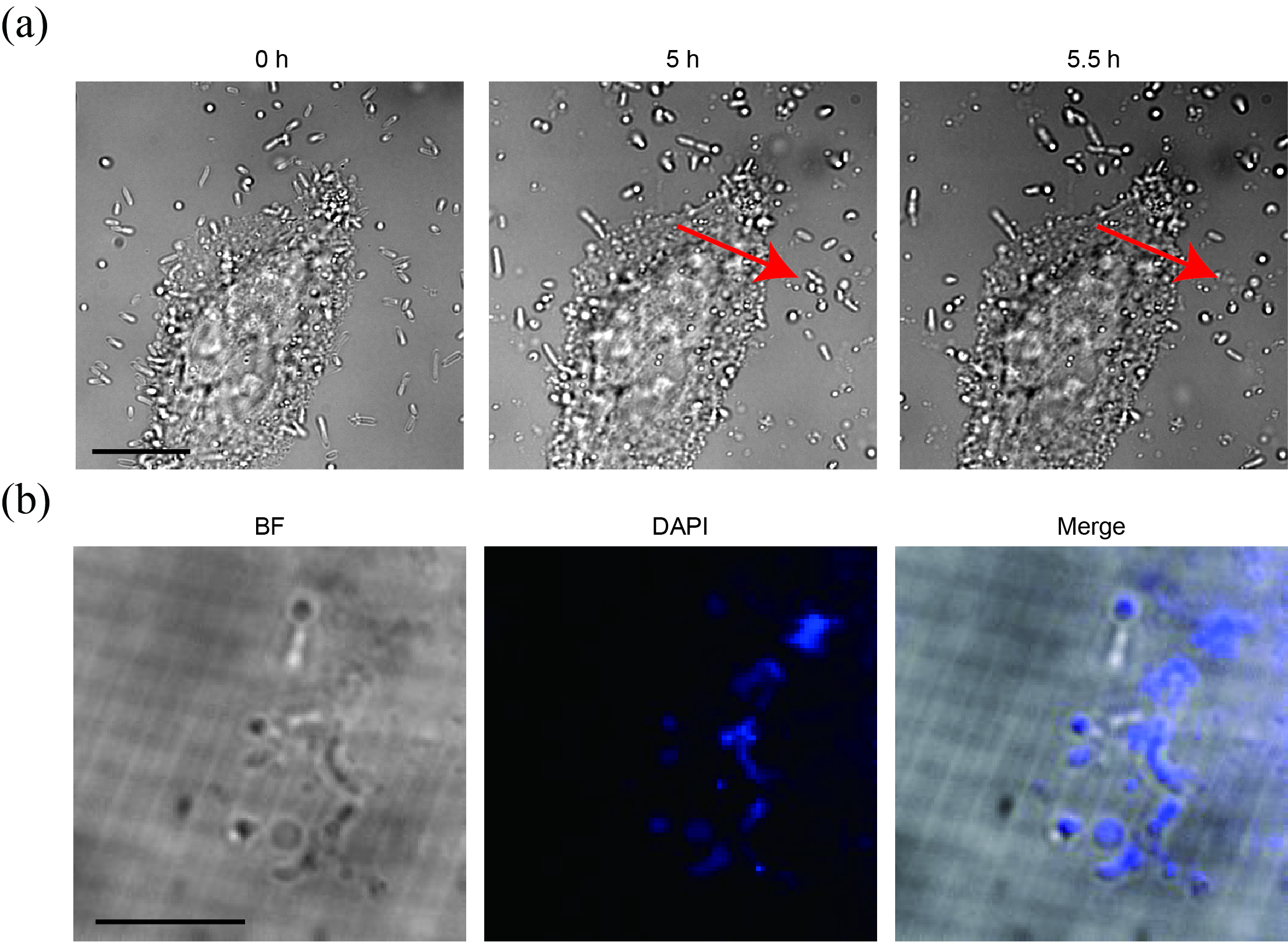

Supplement: nwad031_Supplemental_Files [file nwad031_supplemental_files.zip › supplementary figures/Fig S16.jpg]

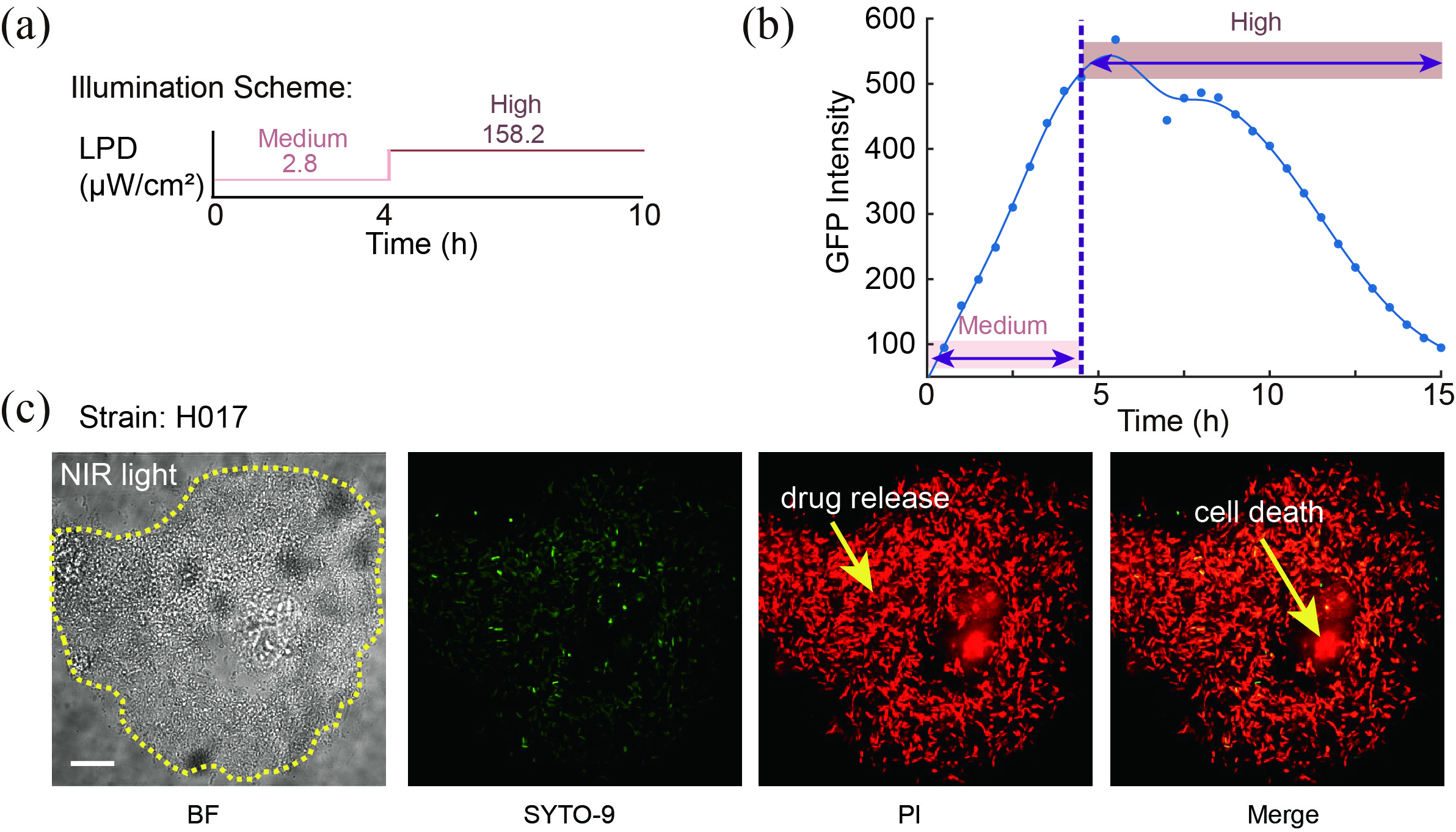

Supplement: nwad031_Supplemental_Files [file nwad031_supplemental_files.zip › supplementary figures/Fig S17.jpg]

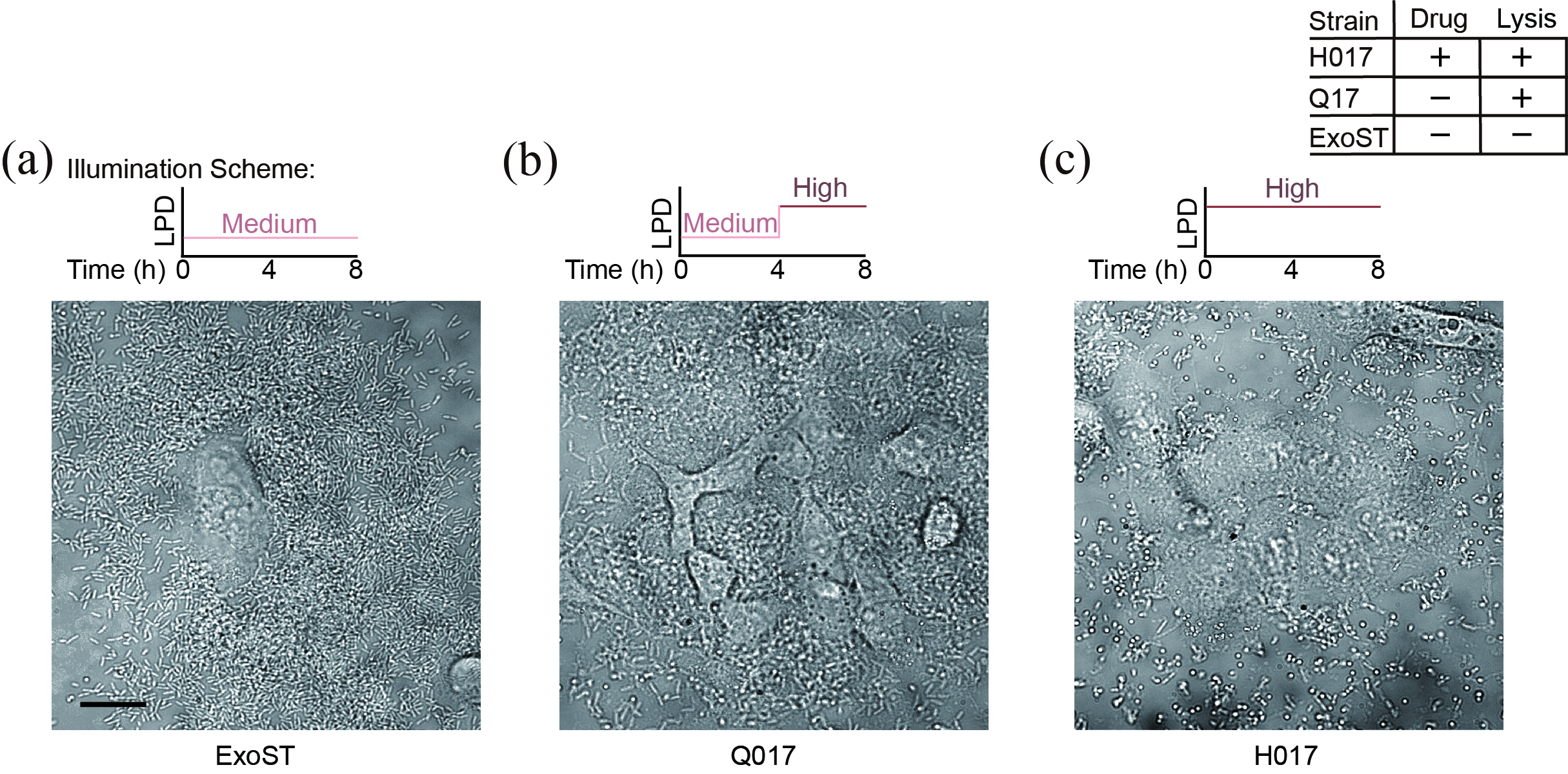

Supplement: nwad031_Supplemental_Files [file nwad031_supplemental_files.zip › supplementary figures/Fig S18.jpg]

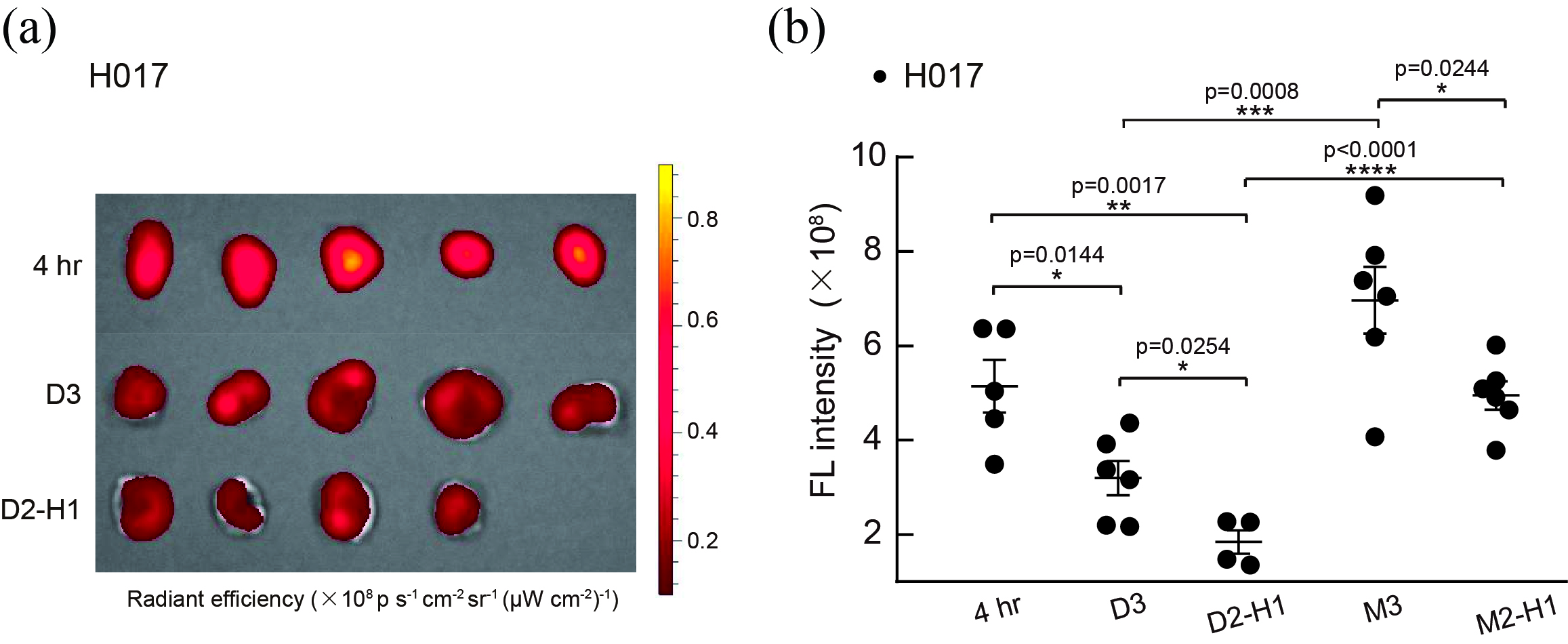

Supplement: nwad031_Supplemental_Files [file nwad031_supplemental_files.zip › supplementary figures/Fig S19.jpg]

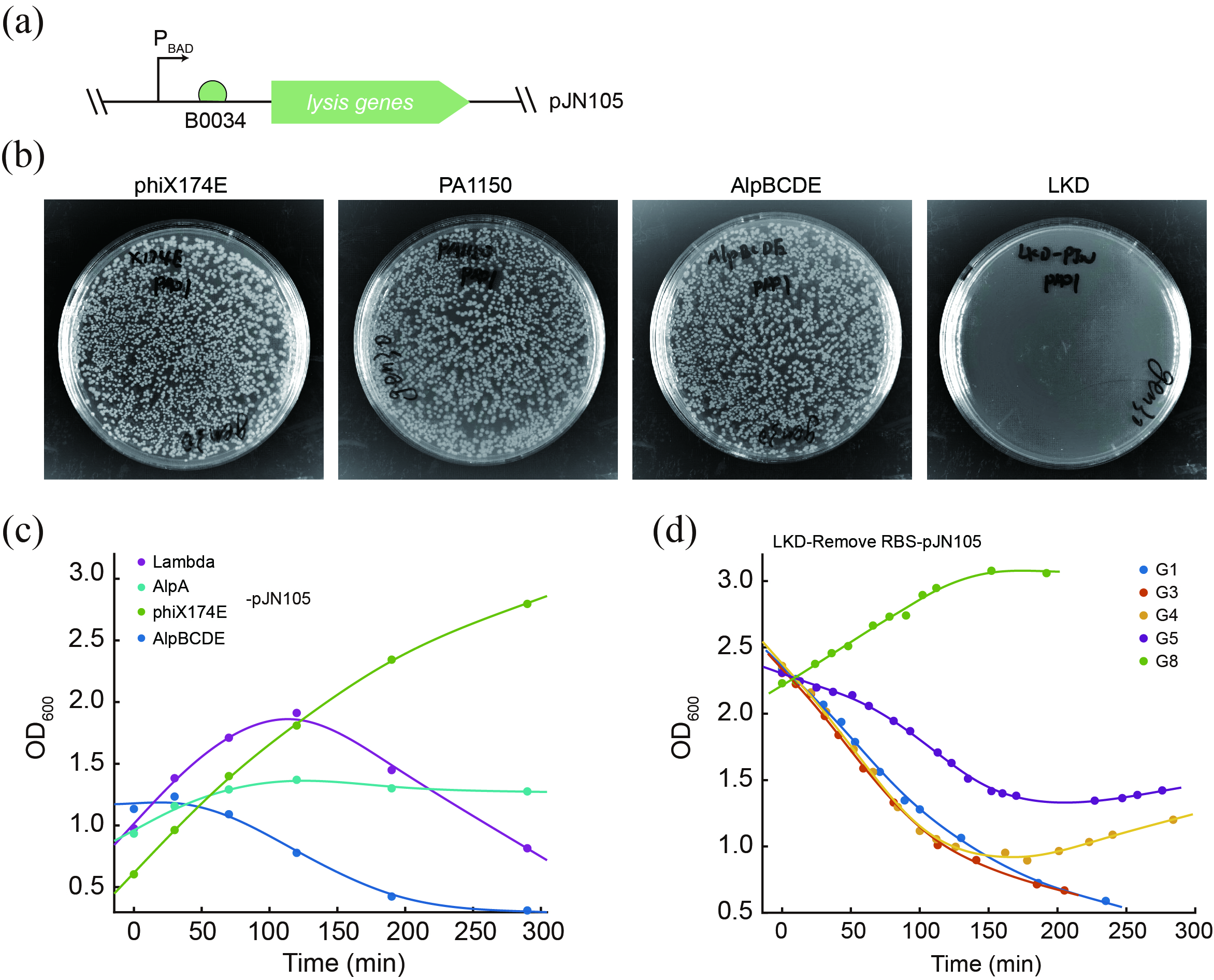

Supplement: nwad031_Supplemental_Files [file nwad031_supplemental_files.zip › supplementary figures/Fig S2.jpg]

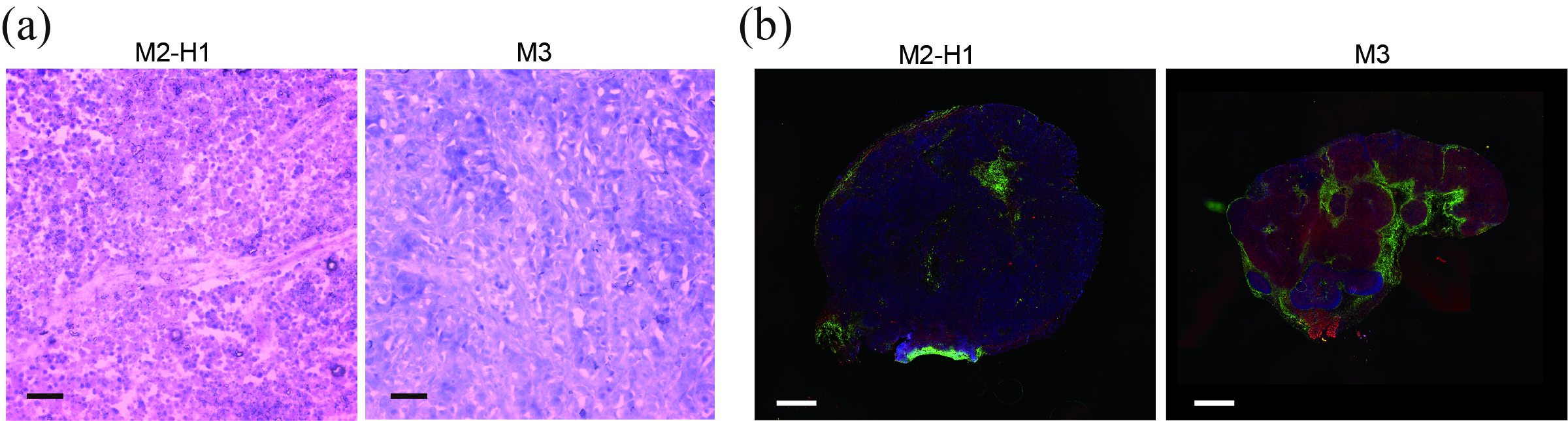

Supplement: nwad031_Supplemental_Files [file nwad031_supplemental_files.zip › supplementary figures/Fig S20.jpg]

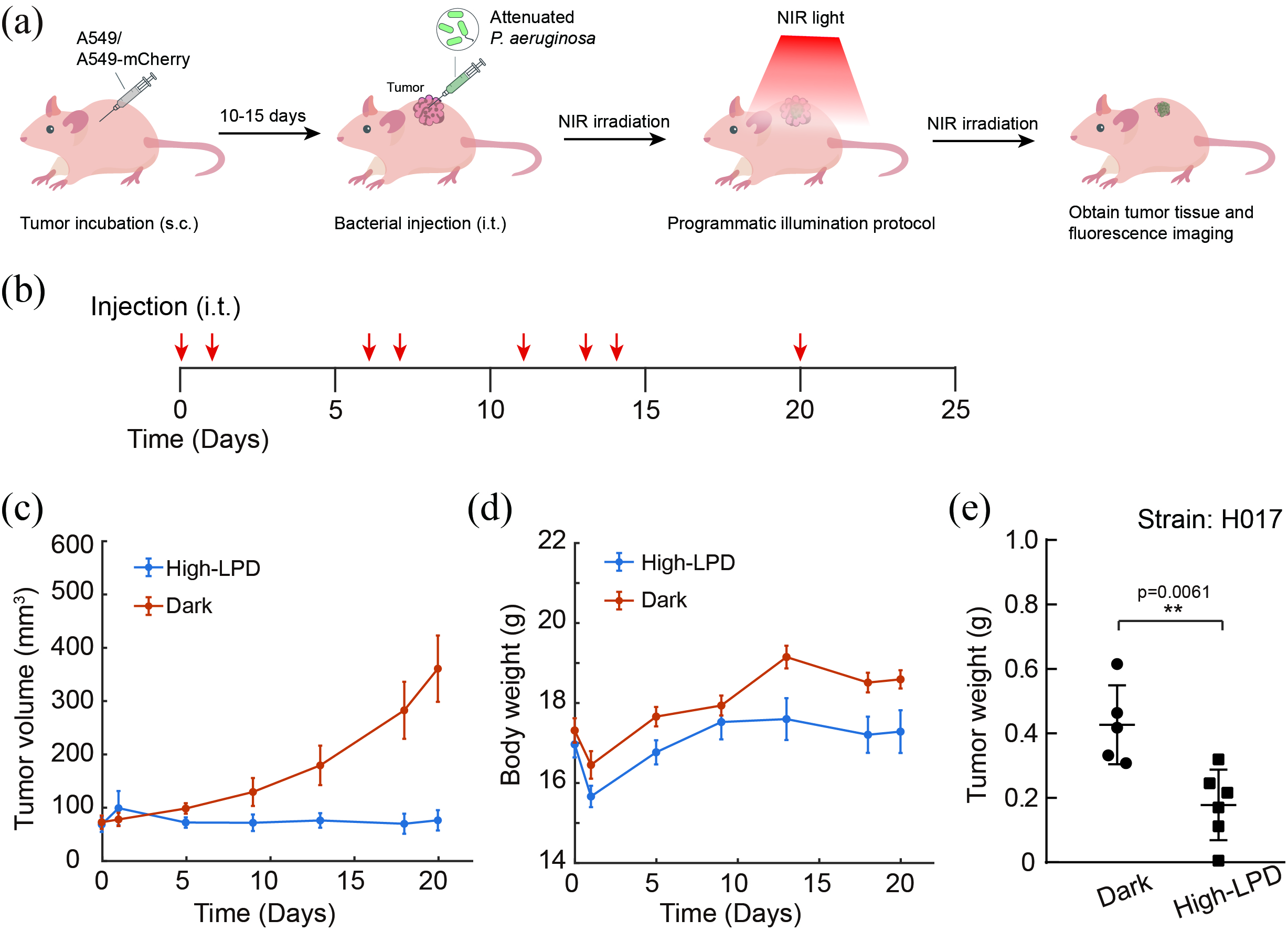

Supplement: nwad031_Supplemental_Files [file nwad031_supplemental_files.zip › supplementary figures/Fig S21.jpg]

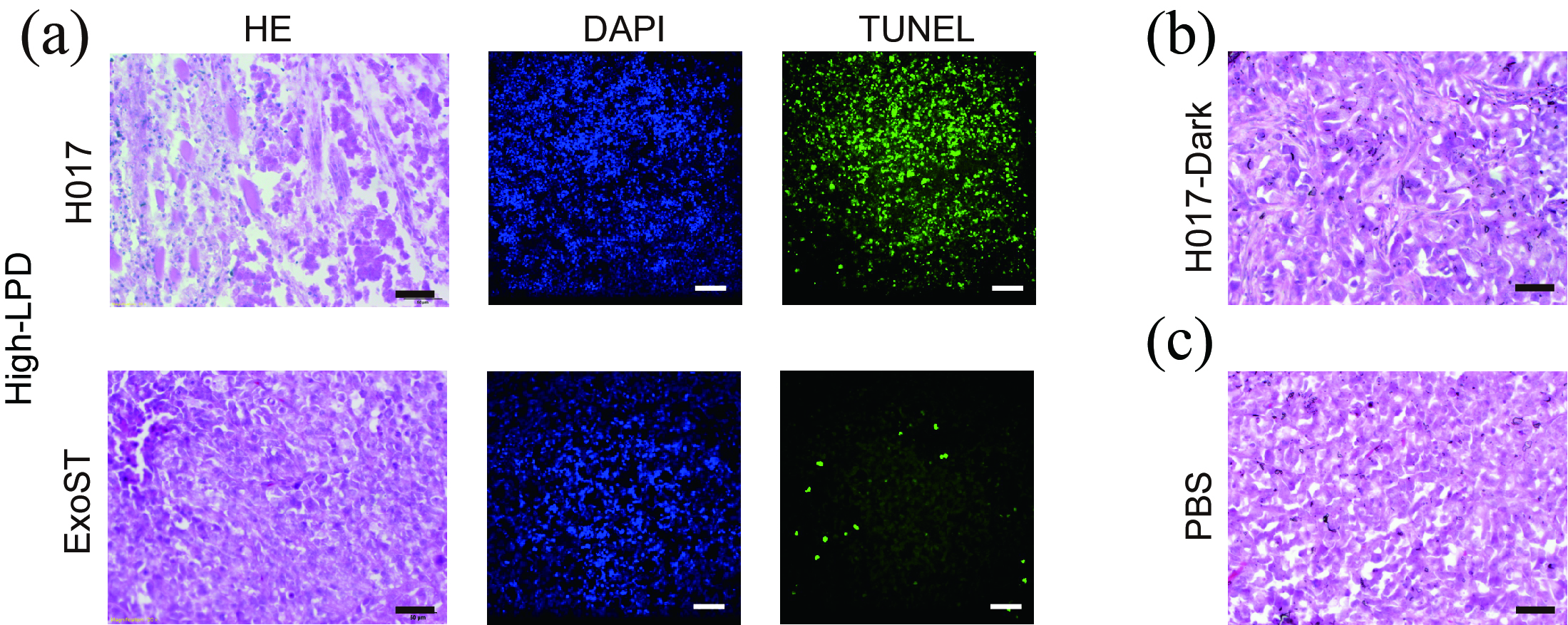

Supplement: nwad031_Supplemental_Files [file nwad031_supplemental_files.zip › supplementary figures/Fig S22.jpg]

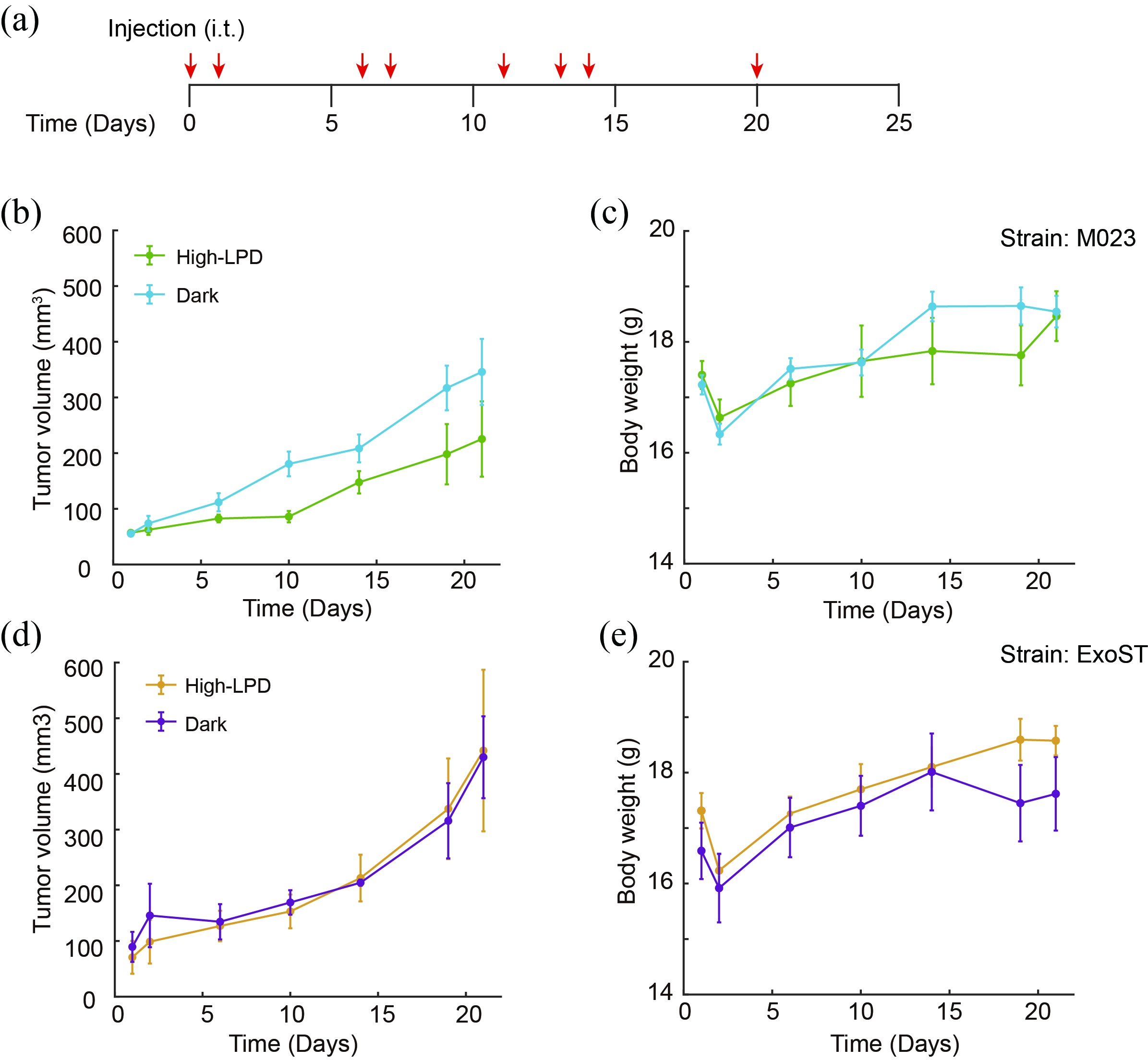

Supplement: nwad031_Supplemental_Files [file nwad031_supplemental_files.zip › supplementary figures/Fig S23.jpg]

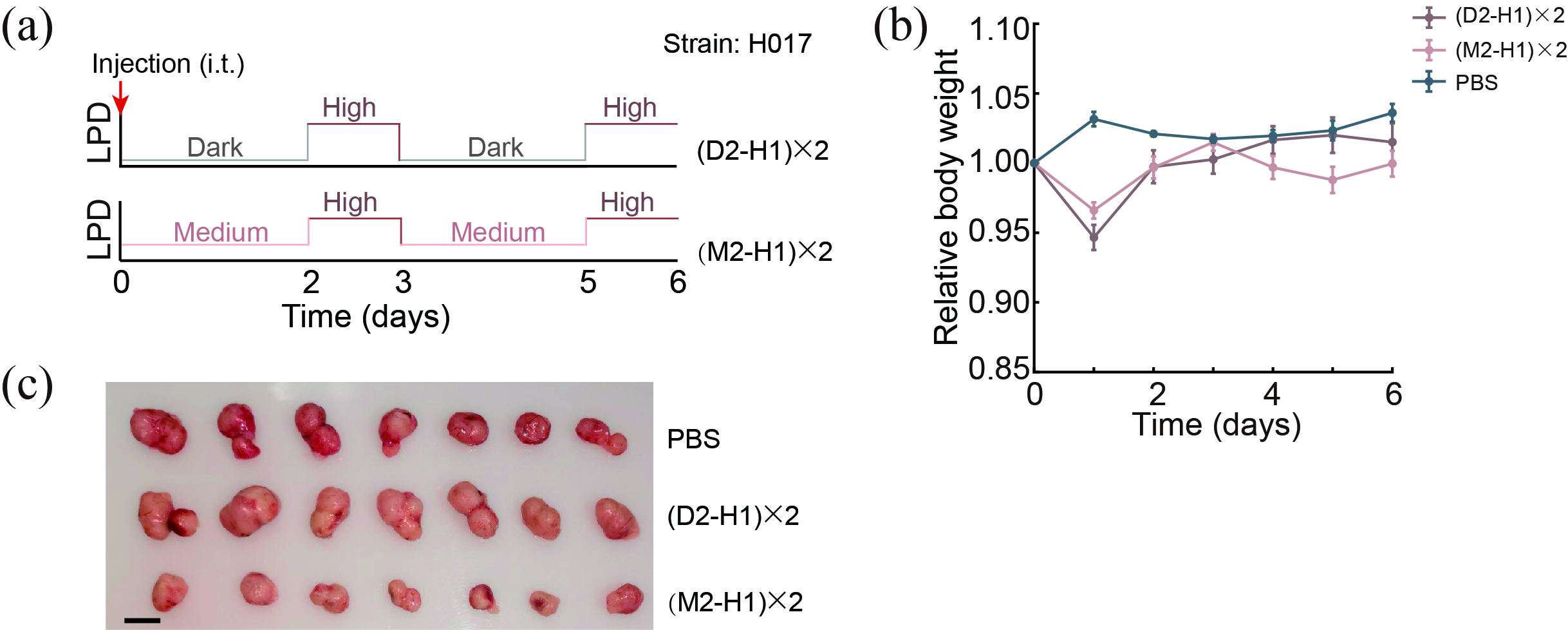

Supplement: nwad031_Supplemental_Files [file nwad031_supplemental_files.zip › supplementary figures/Fig S24.jpg]

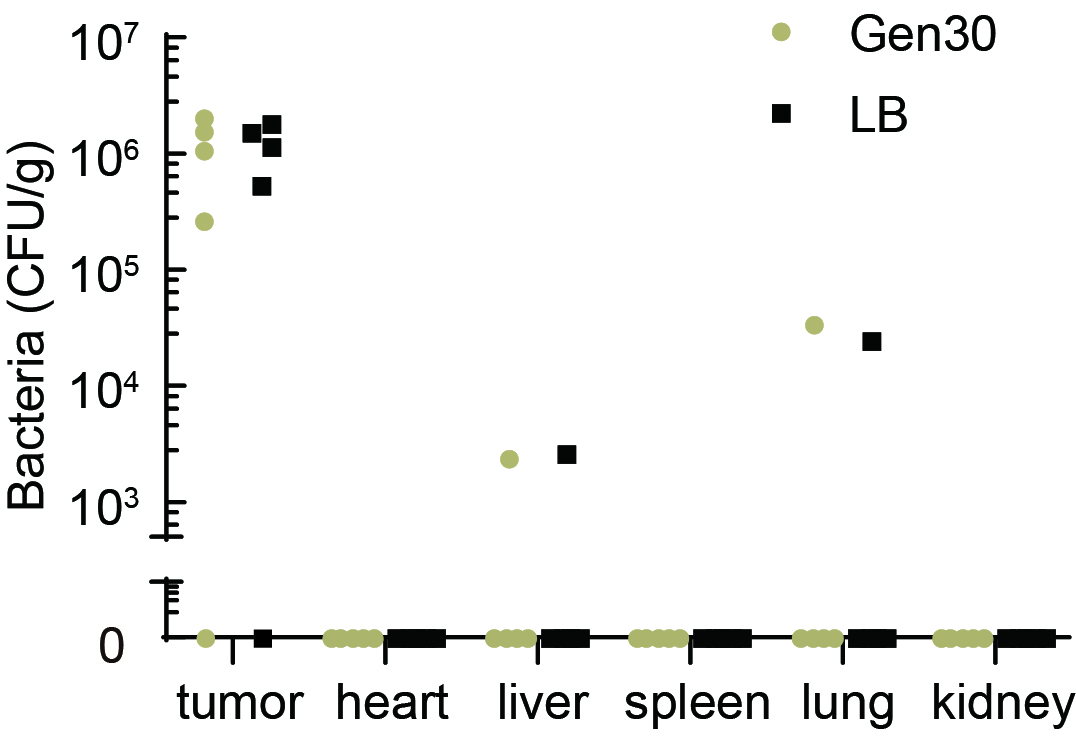

Supplement: nwad031_Supplemental_Files [file nwad031_supplemental_files.zip › supplementary figures/Fig S25.jpg]

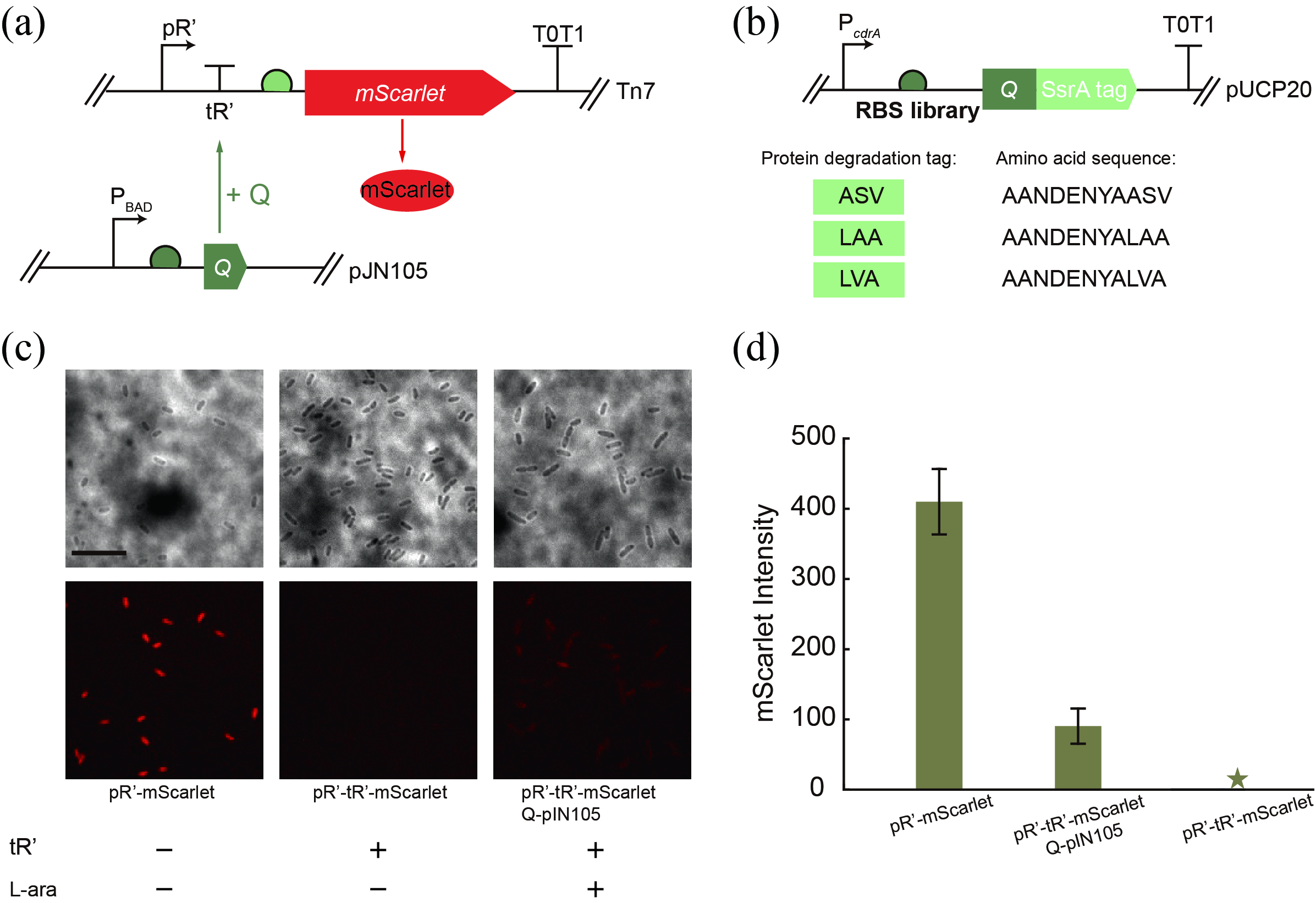

Supplement: nwad031_Supplemental_Files [file nwad031_supplemental_files.zip › supplementary figures/Fig S3.jpg]

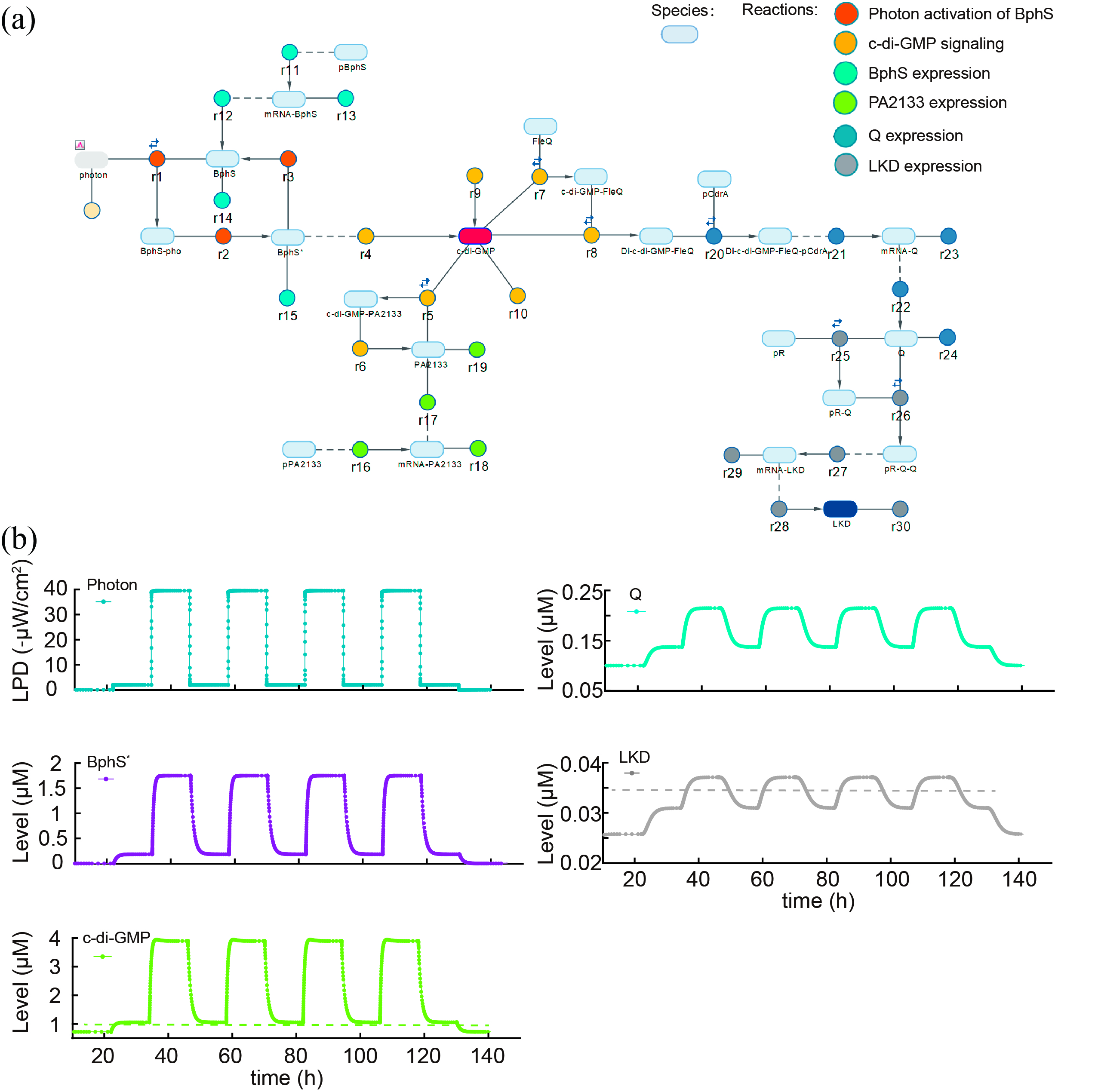

Supplement: nwad031_Supplemental_Files [file nwad031_supplemental_files.zip › supplementary figures/Fig S4.jpg]

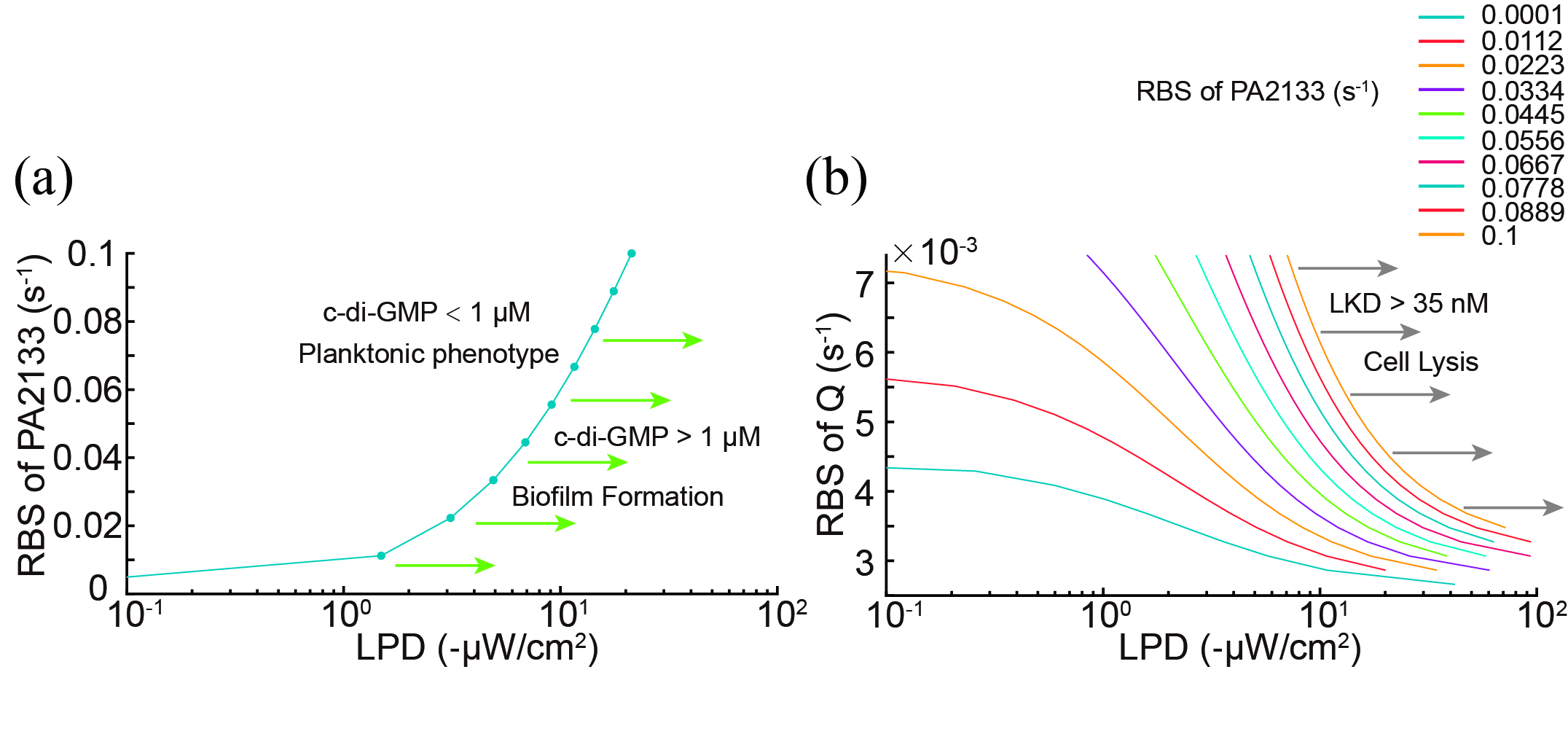

Supplement: nwad031_Supplemental_Files [file nwad031_supplemental_files.zip › supplementary figures/Fig S5.jpg]

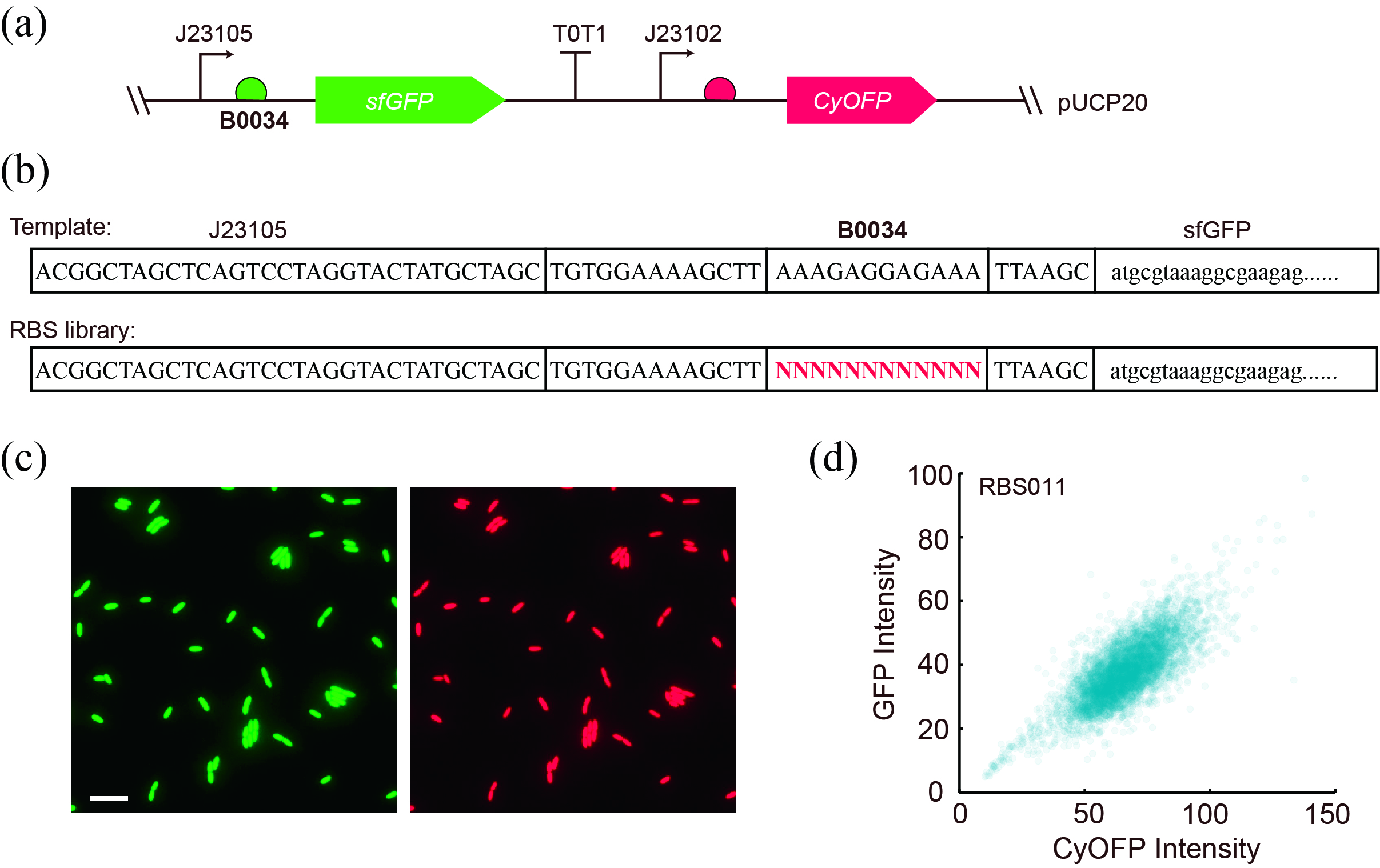

Supplement: nwad031_Supplemental_Files [file nwad031_supplemental_files.zip › supplementary figures/Fig S6.jpg]

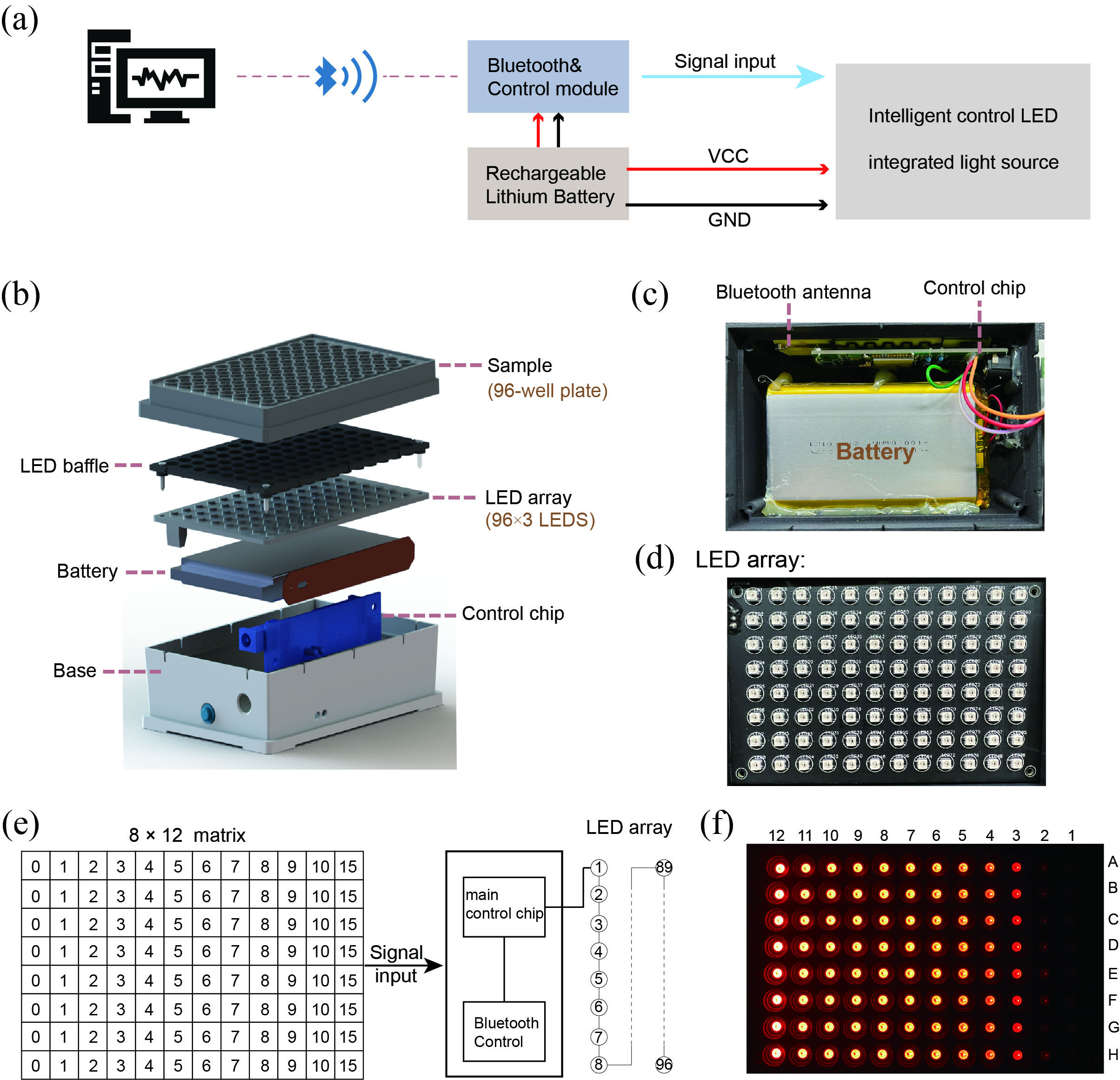

Supplement: nwad031_Supplemental_Files [file nwad031_supplemental_files.zip › supplementary figures/Fig S7.jpg]

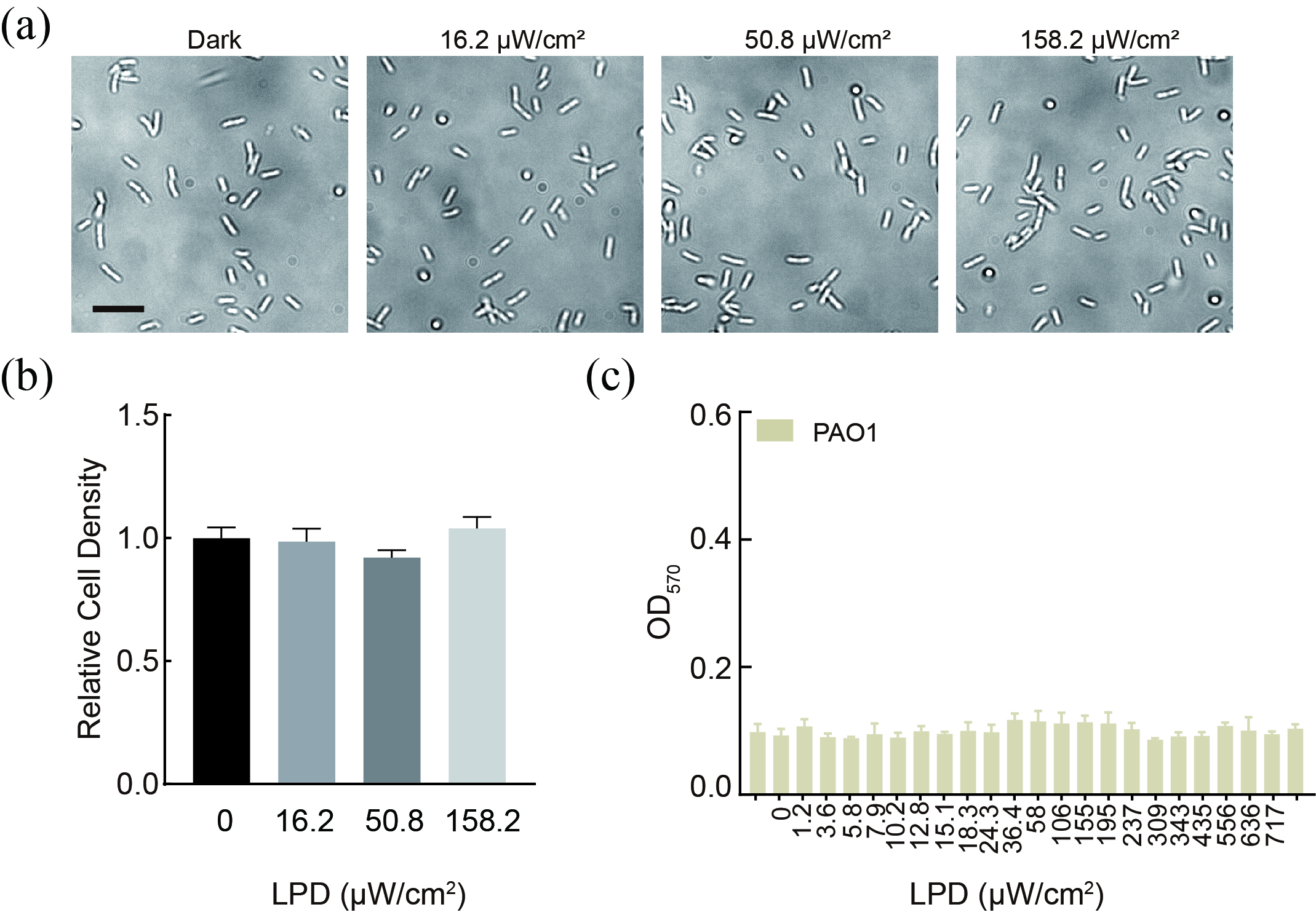

Supplement: nwad031_Supplemental_Files [file nwad031_supplemental_files.zip › supplementary figures/Fig S8.jpg]

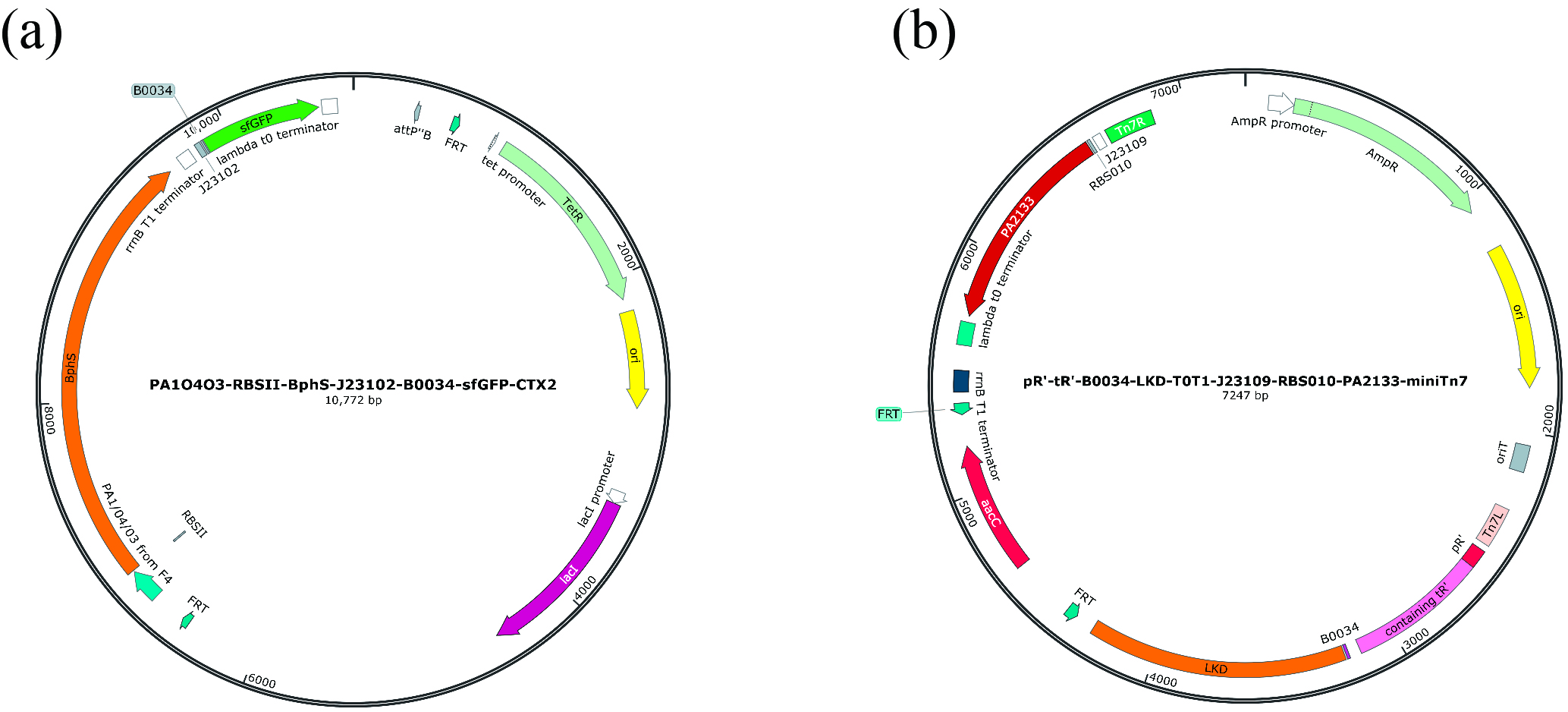

Supplement: nwad031_Supplemental_Files [file nwad031_supplemental_files.zip › supplementary figures/Fig S9.jpg]
